# Asymmetric and symmetric protein arginine methylation in methionine-addicted human cancer cells

**DOI:** 10.1371/journal.pone.0296291

**Published:** 2023-12-22

**Authors:** Ashley G. Holtz, Troy L. Lowe, Yusuke Aoki, Yutaro Kubota, Robert M. Hoffman, Steven G. Clarke

**Affiliations:** 1 Department of Chemistry and Biochemistry, University of California, Los Angeles, Los Angeles, CA, United States of America; 2 AntiCancer, Inc, San Diego, CA, United States of America; 3 Department of Surgery, University of California, San Diego, La Jolla, CA, United States of America; 4 Department of Orthopedic Surgery, Graduate School of Medicine, University of the Ryukyus, Nishihara, Japan; UCL Institute of Neurology: UCL Queen Square Institute of Neurology, UNITED KINGDOM

## Abstract

The methionine addiction of cancer cells is known as the Hoffman effect. While non-cancer cells in culture can utilize homocysteine in place of methionine for cellular growth, most cancer cells require exogenous methionine for proliferation. It has been suggested that a biochemical basis of this effect is the increased utilization of methionine for *S*-adenosylmethionine, the major methyl donor for a variety of cellular methyltransferases. Recent studies have pointed to the role of *S*-adenosylmethionine-dependent protein arginine methyltransferases (PRMTs) in cell proliferation and cancer. To further understand the biochemical basis of the methionine addiction of cancer cells, we compared protein arginine methylation in two previously described isogenic cell lines, a methionine-addicted 143B human osteosarcoma cell line and its less methionine-dependent revertant. Previous work showed that the revertant cells were significantly less malignant than the parental cells. In the present study, we utilized antibodies to detect the asymmetric dimethylarginine (ADMA) and symmetric dimethylarginine (SDMA) products of PRMTs in polypeptides from cellular extracts and purified histone preparations of these cell lines fractionated by SDS-PAGE. Importantly, we observed little to no differences in the banding patterns of ADMA- and SDMA-containing species between the osteosarcoma parental and revertant cell lines. Furthermore, enzymatic activity assays using *S*-adenosyl-ʟ-[methyl-^3^H] methionine, recombinantly purified PRMT enzymes, cell lysates, and specific PRMT inhibitors revealed no major differences in radiolabeled polypeptides on SDS-PAGE gels. Taken together, these results suggest that changes in protein arginine methylation may not be major contributors to the Hoffman effect and that other consequences of methionine addiction may be more important in the metastasis and malignancy of osteosarcoma and potentially other cancers.

## Introduction

Methionine is an essential amino acid whose metabolites are involved in a variety of cellular pathways. Methionine addiction of cancer cells is a phenomenon where cancer cells require exogenous methionine to grow and are unable to survive when methionine in the culture medium is replaced with homocysteine, methionine’s precursor [1–6]. However, paradoxically cancer cells have the ability to produce large amounts of methionine from homocysteine and can use endogenously synthesized methionine for cellular processes [1, 7, 8]. In contrast, normal cells can grow in culture when homocysteine is substituted for methionine [6, 7]. The methionine addiction of cancer cells is known as the Hoffman effect and is a target for cancer treatment [2, 3, 9].

Studies in xenograft mouse models show that treatment with recombinant methioninase, an enzyme that depletes plasma methionine levels, and/or a methionine-restricted diet can decrease tumor volume [10–14]. Clinically, the enhanced usage of methionine in cancer cells can be used to localize tumors with [^11^C]-methionine positron emission tomography (MET-PET), particularly for cancers in the brain as well as organ cancers [15–17]. Additionally, methionine restriction has been used as a combination treatment with chemotherapy in cancer patients [9]. A methionine-restricted diet in Phase I clinical trials as well as a Phase I study of methioninase with cancer patients have been shown to reduce plasma methionine levels without major complications [18, 19]. In initial studies of three patients with metastatic breast cancer, i.v. infusion of methioninase reduced serum methionine levels without any significant side effects [20]. Oral methioninase given to three patients with advanced prostate cancer decreased prostate-specific antigen levels, and has had beneficial effects in several patients with pancreatic cancer, rectal cancer, and breast cancer [21–26]. Overall, methionine restriction appears to be safe for patients and is potentially effective for reducing the severity of cancer.

Methionine is not only used in protein synthesis but also for polyamine metabolism, purine and pyrimidine synthesis, glutathione synthesis, and methylation reactions [27]. Because methionine is required for a wide variety of cellular functions, the exogenous requirement for methionine in cancer cells despite their capacity to synthesize and use methionine endogenously at high levels is incompletely understood [1, 5, 7, 8]. As methionine addiction of cancer is becoming more clinically relevant, it is important to understand how methionine affects cancer progression at a molecular level. To elucidate the molecular mechanism of methionine addiction, Aoki et. al derived a less methionine-dependent revertant cell line from the methionine-addicted human osteosarcoma 143B cell line that demonstrated improved survival at high levels of recombinant methioninase (rMETase) treatment [28]. After culturing the cells in rMETase, methionine was depleted from the culture medium, so most of the cancer cells did not survive. The cells cultured with rMETase that survived after four cycles of selection were designated as less methionine-dependent revertant cells. In cell culture with rMETase, the cell viability of the 143B revertant (143B-R) cells was higher than that of the 143B parent (143B-P) cells [28]. At higher concentrations of rMETase, the 143B-P cells were unable to survive in culture while the 143B-R cells still could grow [28]. Additionally, the morphology between the two cell lines differed, with the 143B-R cells appearing more normal fibroblast-like, larger, and aligned in one direction compared to the 143B-P cells. With a wound-healing assay and a cell-invasion assay, the 143B-R cells showed significantly lower cell migration and invasion ability, respectively, than the 143B-P cells. In orthotopic xenograft mouse models, the 143B-R cells grew smaller tumors than the 143B-P cells and did not metastasize unlike the 143B-P cells. There were no histological differences observed between primary tumors arising from 143B-P and 143B-R cells. The 143B-R cells had increased protein expression of ZO-1, an epithelial marker, and decreased expression of the mesenchymal markers vimentin, Snail, and Slug, compared to the 143B-P cells upon immunoblotting [28]. Taken together, the results of this previous study show that the 143B-R cells are less malignant than their parental cells. Revertant cells of other methionine-addicted cancers have also been shown to lose their malignancy with their methionine dependence [29].

Methionine addiction of cancer cells is due at least in part to elevated transmethylation reactions [5, 29–35]. In the previous study, it was found that the 143B-R cells had differences in histone lysine methylation [28], suggesting that this modification may be one of the links between the simultaneous loss of methionine addiction and malignancy in the 143B-R cells, possibly by changing the expression of important genes for cancer progression. In the present work, we wanted to determine the effect of the reversion of methionine addiction on protein arginine methylation, a methionine-dependent modification that is often affected in cancer [36–45]. Protein arginine methylation is a post-translational modification catalyzed by methyltransferases (PRMTs) that are involved in a variety of biological processes, including transcriptional regulation, the immune response, RNA splicing, and DNA repair [36, 37, 46, 47]. The methyl donor of these enzymes is *S*-adenosylmethionine (AdoMet), a direct product of methionine. In mammals, nine PRMTs have been identified and are categorized by the products that they form. While all PRMTs initially form monomethylarginine (MMA), Type I PRMTs (PRMTs 1–4, 6, and 8) produce asymmetric dimethylarginine (ADMA) and Type II PRMTs (PRMTs 5 and 9) produce symmetric dimethylarginine (SDMA) [47]. PRMT7 is a Type III enzyme, which only forms MMA [47, 48]. PRMT1 and PRMT5 are the major Type I and Type II enzymes and are responsible for the majority of ADMA and SDMA marks on proteins, respectively [49, 50]. Of the three types of protein arginine methylation, ADMA is the most abundant in cells, followed by SDMA and then MMA [51]. Significantly, the overexpression of PRMTs in cancer is often linked to poor prognosis [36, 37, 39, 40, 42, 44, 45], and PRMT inhibitors, especially against PRMT5, are currently being tested in clinical trials with cancer patients [52–56]. Because the formation of AdoMet requires methionine and PRMTs are commonly upregulated in cancer, we hypothesized that cancer cells would more extensively utilize methionine in the biosynthesis of AdoMet and in PRMT-dependent methylation reactions of cellular proteins. To test this hypothesis, we wanted to compare protein arginine methylation levels in the previously described methionine-addicted parent and less methionine-dependent revertant cell lines.

Using antibodies against ADMA and SDMA in immunoblots, we show that the 143B-P and 143B-R cells had no major differences in the level of ADMA or SDMA on proteins in cellular extracts and on purified histones. Additionally, enzymatic activity assays with *S*-adenosyl-ʟ-[methyl-^3^H] methionine ([^3^H]-AdoMet), recombinantly purified PRMT1, 5, and 7 enzymes, and intracellular substrates showed a similar banding pattern of radiolabeled polypeptides between the parent and revertant cells. Overall, no significant changes in PRMT activity were observed in the revertant cells, suggesting that the involvement of PRMTs in cancer may be through a mechanism distinct from the Hoffman effect.

## Results

### Revertant human 143B osteosarcoma cells are less sensitive to methionine restriction with recombinant methioninase

**Fig 1:**
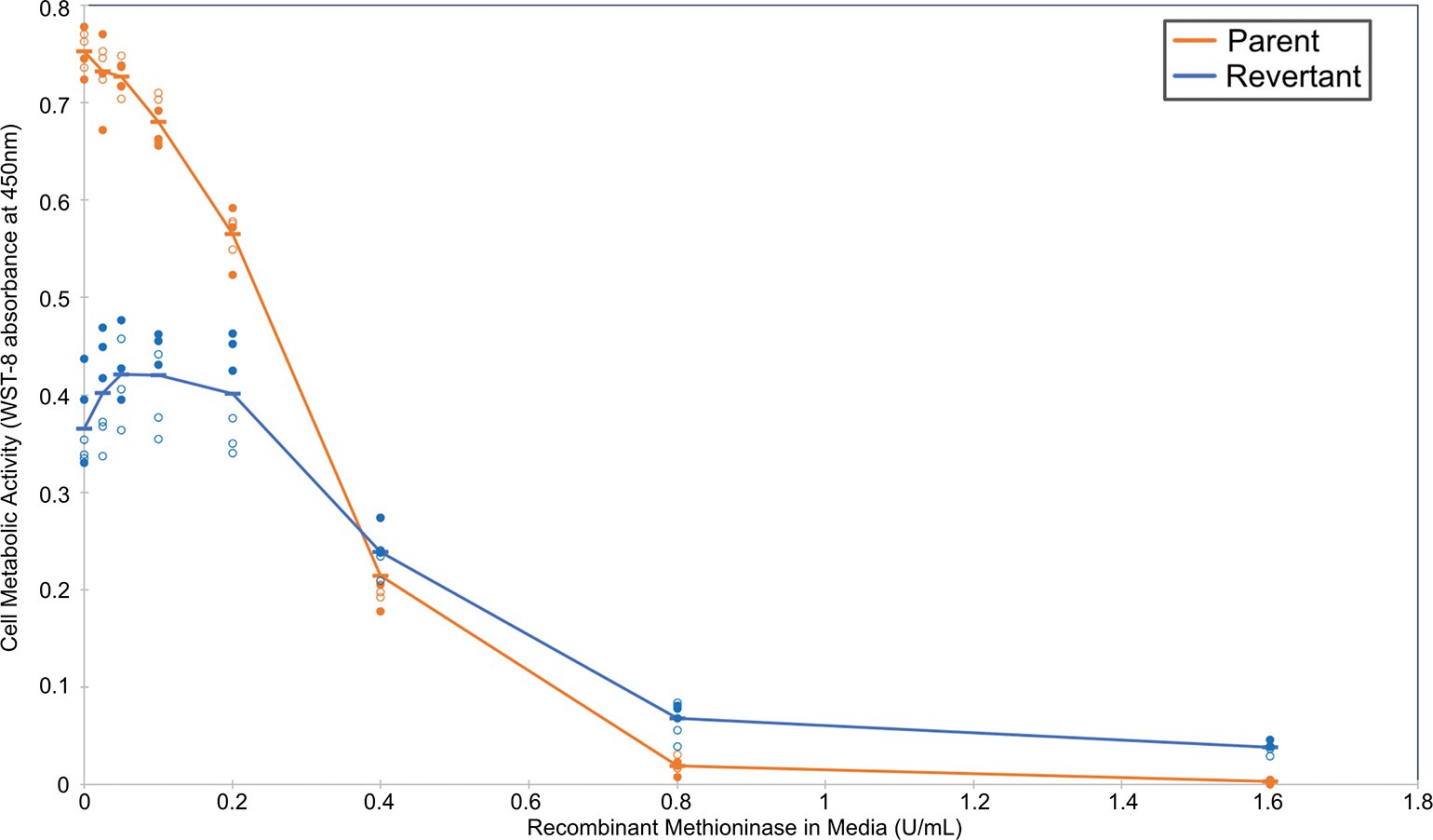
Analysis of methionine dependence in human osteosarcoma 143B parent cells and in revertant cells grown on low-methionine media.

We first analyzed whether the 143B-P and 143B-R cells responded differently to methionine restriction with rMETase. The revertant cells had greater cell viability at high concentrations of rMETase concentrations compared to their parental cells, indicating their relative independence from methionine, consistent with previous results [28] (Fig 1). The IC_50_ of rMETase was 0.46 U/mL in the revertant cells and 0.29 U/mL in the parental cells. Therefore, we would possibly see the effects of different degrees of methionine dependence on *S*-adenosylmethionine-dependent methyltransferases, including those involved in protein arginine methylation. Thus, we began to explore how the difference in methionine dependence between the parental and revertant cells may affect the activity of PRMTs.

Cells were grown in normal media and then cultured for three days in media containing various concentrations of recombinant methioninase to lower the methionine level, as previously described [28]. Cell viability was measured in an assay using water-soluble tetrazolium-8 (WST-8) as previously described [28]. The absorbance at 450 nm after subtracting a no-cell blank is indicated on the y-axis. The results represent two separate experiments performed in triplicate. The closed circles represent one experiment and the open circles represent a second individual experiment following the same procedure. The measurements from the parent 143B are shown in orange, and those from the revertant 143B cells are shown in blue. The average values are denoted by a dash and connected by the line.

### The 143B-P and 143B-R cells have no major differences in the levels of proteins marked with ADMA or SDMA

We employed polyclonal antibodies raised against synthetic peptides containing SDMA and ADMA within glycine rich regions [57] to mimic the common substrate sites recognized by PRMT1, -3, -5, -6, and -8 [36, 50, 58]. SDMA is predominantly produced by PRMT5, and ADMA is predominantly produced by PRMT1. We found that neither the anti-SDMA nor the anti-ADMA antibodies had specific reactivity against negative controls compared to mammalian cell lysates and histone fractions, which were used as positive controls (S1 Fig). Negative controls included an unmethylated protein with a glycine and arginine rich region and *Escherichia coli* (*E*. *coli*) lysate, which does not contain PRMTs [59].

**Fig 2:**
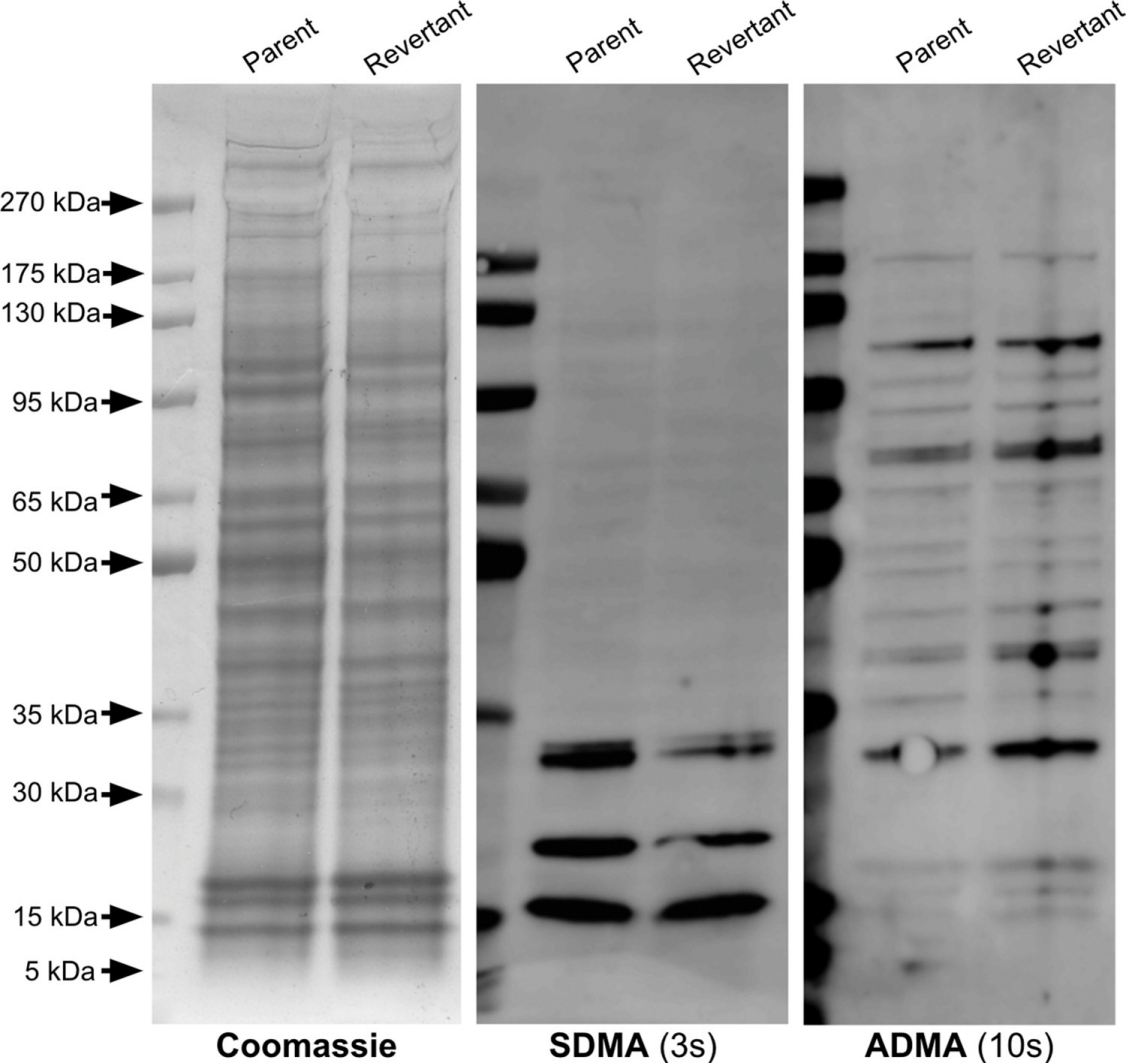
Identification of proteins containing SDMA and ADMA in human parent and revertant 143B osteosarcoma cells.

Next, we explored differences in protein arginine methylation, specifically ADMA and SDMA, in the parent and revertant cancer cell lines. Whole cell extracts of the 143B-P and 143B-R cells were fractionated onto a sodium dodecyl-sulfate polyacrylamide gel electrophoresis (SDS-PAGE) gel, and Coomassie staining was performed as a loading control to compare ADMA and SDMA levels between 143B-P and 143B-R cells (Fig 2). Significantly, no differences in the polypeptide Coomassie staining pattern were observed in two independent experiments, suggesting that there were no major differences in protein expression in the parent and revertant cells (Fig 2). Three proteins with molecular weights of near 30 kDa, 20 kDa, and 15 kDa had an SDMA modification and were at a similar intensity level between the 143B-P and 143B-R cell extracts. In both cell lines, multiple proteins with ADMA modifications are present between approximately 175 kDa and 15 kDa at similar intensity levels. These results indicate that no observable changes in protein arginine methylation accompany the reversion from the methionine-addicted and metastatic phenotype of the parent cells.

The leftmost panel shows a Coomassie-stained SDS-PAGE gel. The middle panel shows an anti-SDMA immunoblot with a 3 second exposure time. The rightmost panel shows an anti-ADMA immunoblot with a 10 second exposure time. Molecular weight markers are shown at the left in the Coomassie-stained gel and as fluorescent bands on the left margin of the immunoblots.

The proteins with ADMA and with SDMA near 15 kDa could represent methylated arginine on histones. Multiple studies have shown the importance of histone arginine methylation in transcriptional controls in cancer cells [37, 60, 61]. While we established that there were no large-scale differences in overall protein arginine methylation, it was difficult to determine whether there were differences in arginine methylation on histones since the whole cell extract contained histones as well as other low-molecular weight cellular proteins. Because histone modifications can affect gene expression, which may affect the mechanism of reversion, we next investigated whether there were observable differences in SDMA and ADMA on histones in the 143B-P and 143B-R cells.

**Fig 3:**
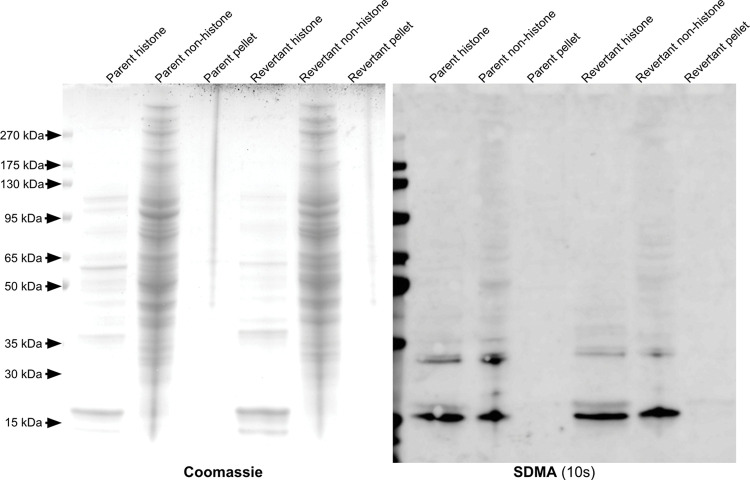
Identification of SDMA-containing polypeptides in histone and non-histone fractions of parent and revertant human osteosarcoma 143B cells.

**Fig 4:**
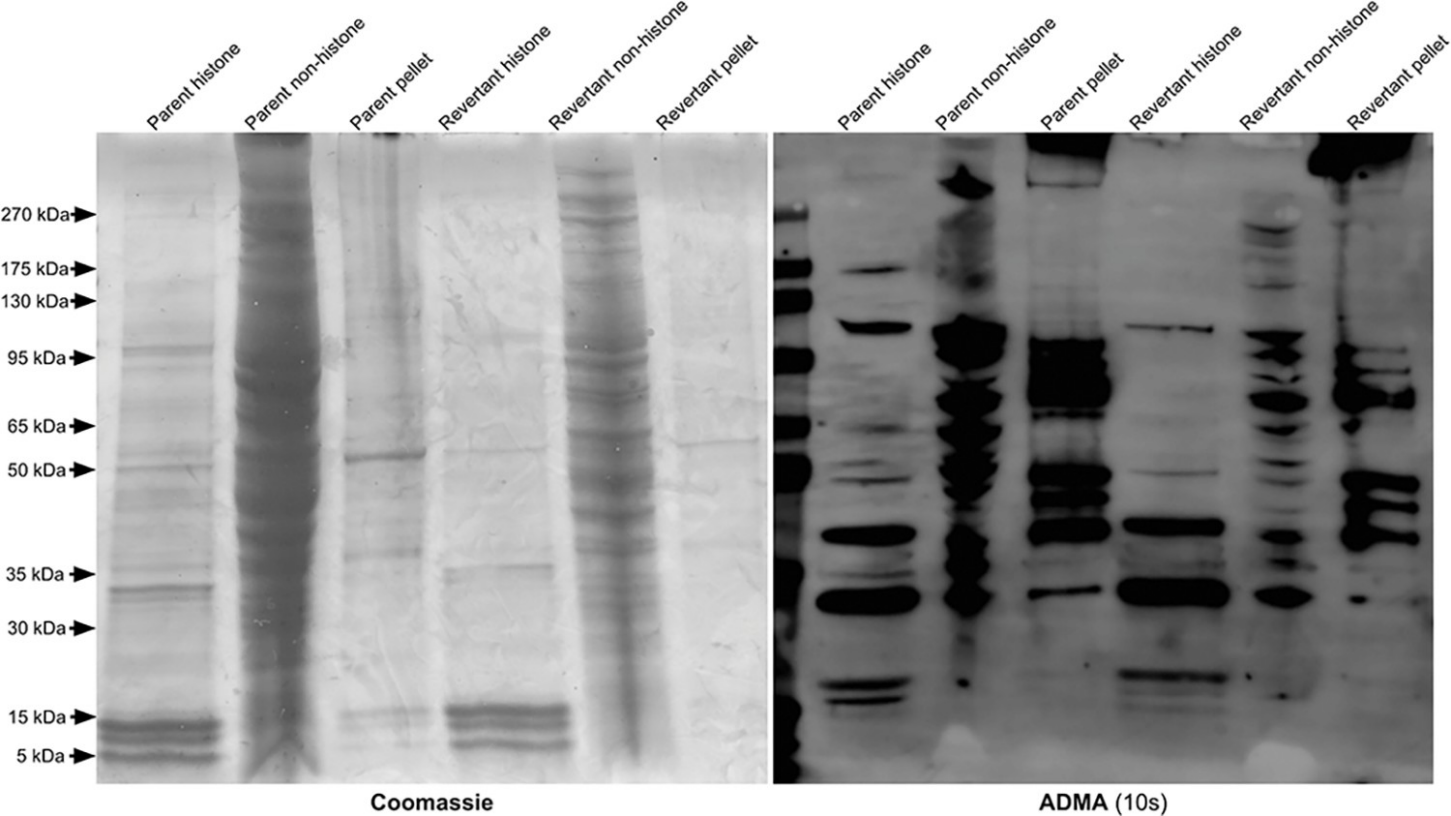
Identification of ADMA-containing polypeptides in histone and non-histone fractions of parent and revertant human osteosarcoma 143B cells.

Extracted histones were analyzed with SDS-PAGE and used in immunoblots against SDMA and ADMA (Fig 3). In the SDMA immunoblot, a band appears slightly above 15kDa in the histone fractions of the 143B-P and 143B-R cells but is not present in the non-histone supernatant, indicating that this protein is a histone. When comparing this histone band between the 143B-P and 143B-R histones, the signal is at a similar intensity, indicating no major differences in SDMA on histones. In the ADMA immunoblot, a histone band near 15 kDa appears more strongly in the 143B-P cells than in the 143B-R cells (Fig 4). However, the Coomassie-stained SDS-PAGE gel shows that the histones are slightly more enriched in the 143B-P cells than in the 143B-R cells, which may reflect the higher level of ADMA on histones of 143B-P cells. Additionally, a difference in ADMA in this molecular weight region was not observed in replicate anti-ADMA immunoblots of cell lysates (S2 Fig). Therefore, we did not find any large-scale differences in ADMA on histones between the 143B-P and 143B-R cells.

The leftmost panel shows a Coomassie-stained SDS-PAGE gel, and the rightmost panel shows an anti-SDMA immunoblot with an exposure time of 10 seconds.

The leftmost panel shows a Coomassie-stained SDS-PAGE gel, and the rightmost panel shows an anti-ADMA immunoblot with an exposure time of 10 seconds.

### The 143B-P and 143B-R cells share similar substrates for PRMT1, PRMT5, and PRMT7

**Fig 5:**
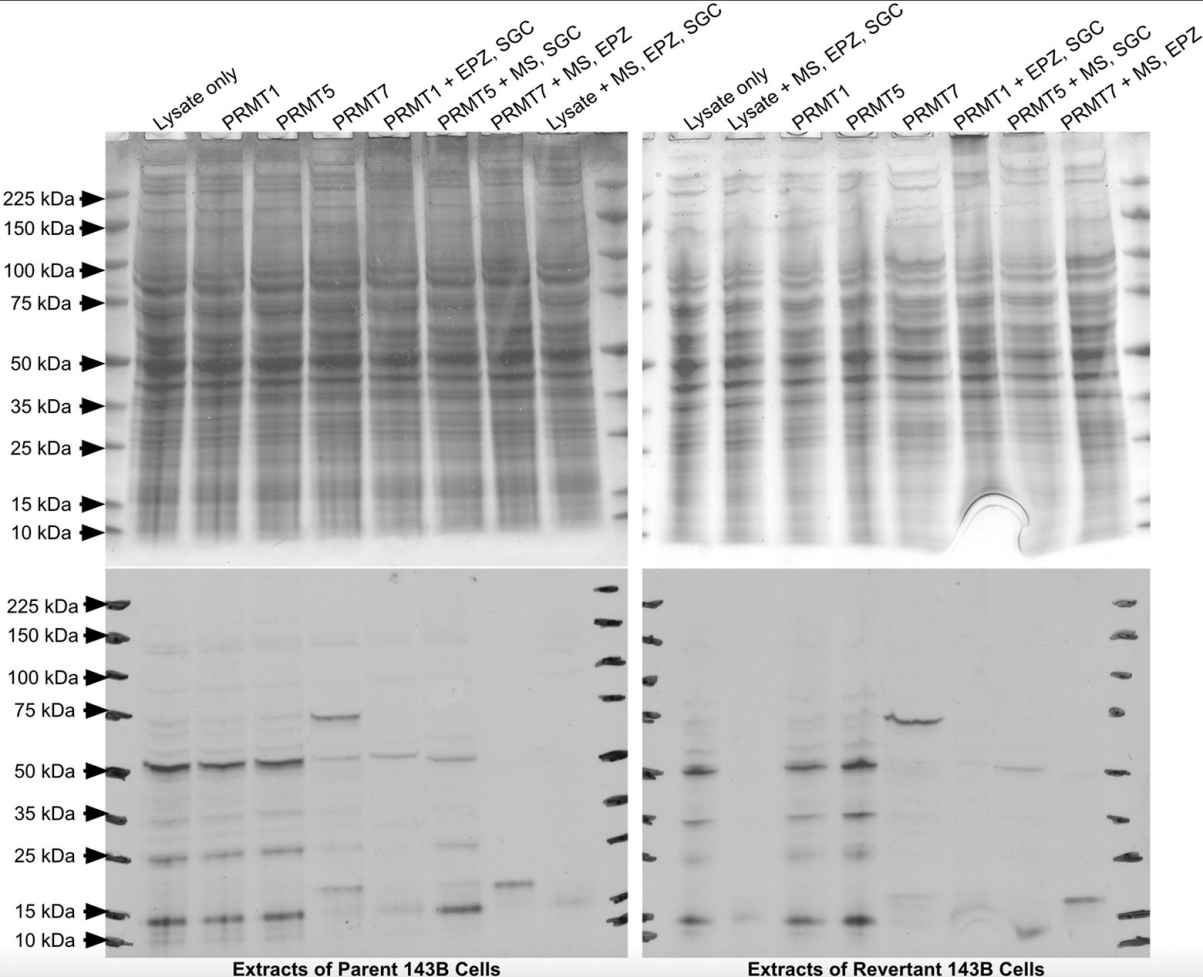
In vitro methylation of polypeptides from extracts of human parent and revertant osteosarcoma 143B cells.

We next performed in vitro enzymatic assays to examine whether there was a difference in substrate availability for PRMTs within the 143B-P and 143B-R cell lysates. Reactions were set up by incubating either the 143B-P or 143B-R cell lysates with [^3^H]-AdoMet, recombinantly purified PRMT1, -5, or -7, and PRMT inhibitors. Highly selective PRMT inhibitors, including the PRMT7 inhibitor SGC8158, the PRMT5 inhibitor EPZ015666, and the Type I PRMT inhibitor MS023, were used to parse out the activity of individual classes of PRMTs. For example, using PRMT5 along with the inhibitors SGC8158 and MS023 should show methylation in lysates due to PRMT5 activity. Separation of the polypeptides within the reaction mixtures by SDS-PAGE was followed by fluorography. In both the 143B-P and 143B-R lysates incubated only with [^3^H]-AdoMet, there are two major bands indicating methylated proteins near 50 kDa and 15 kDa, as well as two minor bands near 35 kDa and 25 kDa (Fig 5). These four bands remain present in the lysates with PRMT1 and PRMT5 added, but no additional bands are shown that would indicate additional methylation by the PRMT enzymes. All four of these major bands decrease upon addition of the PRMT inhibitors. When PRMT5 is added with MS023 and SGC8158, it appears that there is less signal intensity in the revertant cell lysates than in the parent cell lysates, indicating that there may be less substrate availability for PRMT5 in the revertant cell lysates. However, the 50 kDa and 15 kDa proteins appear in both cell lysates under these conditions, indicating that these proteins may be PRMT5 substrates. A protein with SDMA near 15 kDa was also detected in the 143B lysates in Fig 2. Interestingly, when PRMT7 was added with Type I and PRMT5 inhibitors, these four bands were not present while a single band near 20 kDa appeared. PRMT7 is unique from other PRMTs because it is optimally active at the nonphysiological temperature of 15°C [62]. The band observed near 20 kDa in both 143B-P and 143B-R lysates with PRMT7 added may be a PRMT7 substrate. However, the reactions with PRMT7 were performed at 20°C, so this substrate may not be methylated within a cell at 37°C. While there may be slightly less substrates methylated by PRMT5 in the revertant cells, no major differences in substrate availability for the PRMT1, PRMT5, and PRMT7 enzymes were observed between the 143B-P and 143B-R cells. Therefore, these results strengthen our conclusion from the immunostaining experiments shown in Figs 2–4 that protein arginine methylation is unlikely to play a major role in the reversion of the 143B cells following methionine restriction with recombinant methioninase. We note, however, that the parent and revertant cells were grown in high-methionine medium that may mask differences in the expression of PRMTs.

Cell lysates were incubated for one hour with 0.14 μM [^3^H]-AdoMet, recombinant PRMTs (0.20 μg PRMT1, 0.076 μg PRMT5, or 5.5 μg PRMT7), and their inhibitors (2 μM MS023, 20 μM EPZ015666, or 200 nM SGC8158). The inhibitors are abbreviated with their letters at the top of each lane. The images on the top show Coomassie-stained SDS-PAGE gels, and the images on the bottom show films exposed for fluorography for 63 days. All incubations were carried out at 37°C, except for those with PRMT7 which were carried out at 20°C. To separate the activity of each PRMT, the PRMT enzyme was added in addition to inhibitors of the other types of PRMTs. For example, in order to measure activity mainly from PRMT1, PRMT1 was added along with a PRMT5 inhibitor and a PRMT7 inhibitor.

### A similar constellation of SDMA and ADMA modified polypeptides are seen in various human cancer cell lines, including 143B, H460, HCT116, and HeLa cells

Despite their dysregulation in multiple types of cancer and connection to the methionine metabolic pathway, PRMTs did not seem to heavily contribute to the reversion of the 143B cells to a methionine-independent phenotype. Next, we compared the proteins with SDMA and ADMA found in the bone-derived 143B cells to those in other methionine-addicted cancer cell lines to determine if there are common substrates observed in multiple cancer types. Lung-derived H460 cells and colon-derived HCT116 cells also have the capacity to revert to a less malignant phenotype upon methionine restriction [29], and cervix-derived HeLa cells have been shown to undergo cell cycle arrest when grown in methionine-depleted medium [63].

**Fig 6:**
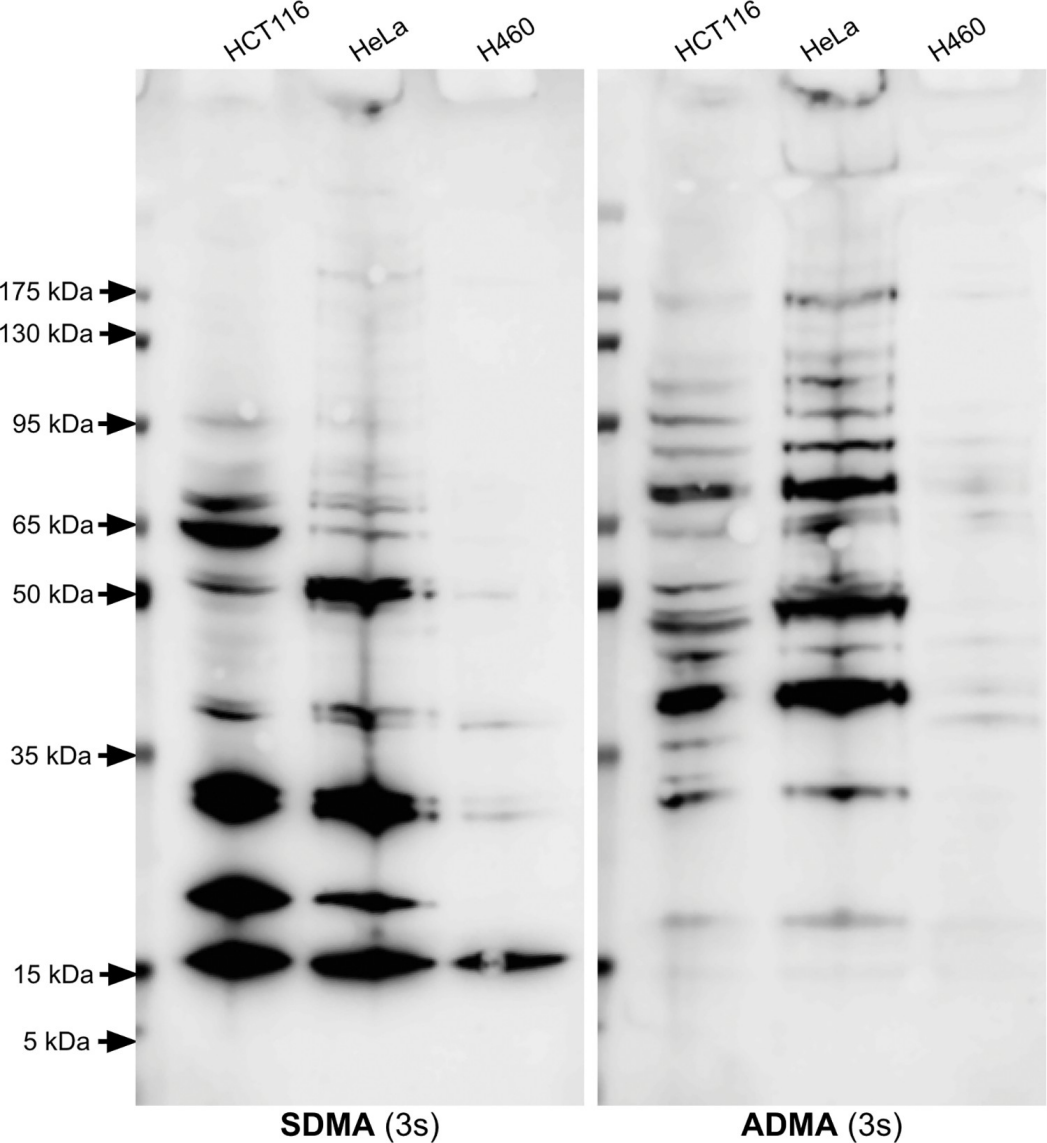
Identification of proteins containing SDMA and ADMA in whole cell extracts of the human colorectal cancer HCT116 cell line, human cervical cancer HeLa cell line, and human non-small cell lung cancer H460 cell line.

Overall, the H460 cells had the lowest amount of protein arginine methylation compared to the 143B, the HCT116, and the HeLa cells (Figs 2 and 6). The 143B cells also had a lower amount of proteins with SDMA compared to the HCT116 and HeLa cells. The HCT116 and HeLa cells showed a very similar banding pattern with both the SDMA and ADMA antibodies. ADMA seemed to be most abundant in proteins above 35 kDa in all four cell lines, while SDMA was most abundant in proteins below 100 kDa. A protein with ADMA slightly above 15 kDa was observed in the HCT116 and HeLa cells. Proteins with SDMA with molecular weights of approximately 15 kDa, 20 kDa, and 30 kDa were observed at a high level in the 143B, HCT116, and HeLa cells, while multiple proteins with ADMA were observed in these three cell lines.

Extracts loaded on the gel represented approximately 250,000 cells. The left panel is an anti-SDMA immunoblot, and the right panel is an anti-ADMA immunoblot. Each has an exposure time of 3 seconds. These images are representative of two individual experiments.

**Fig 7:**
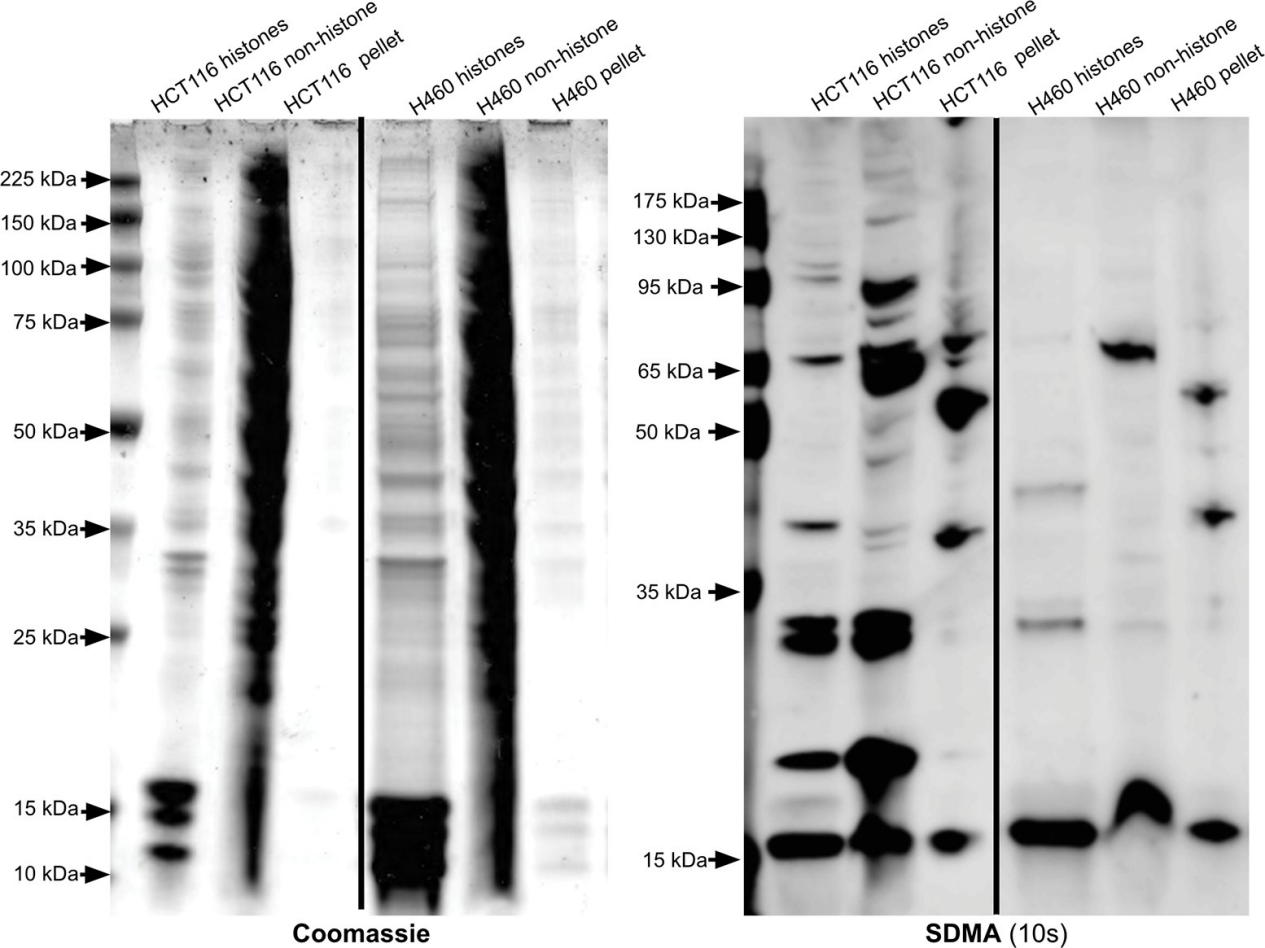
Identification of SDMA-containing polypeptides in histone and non-histone fractions of HCT116 and H460 cell extracts.

**Fig 8:**
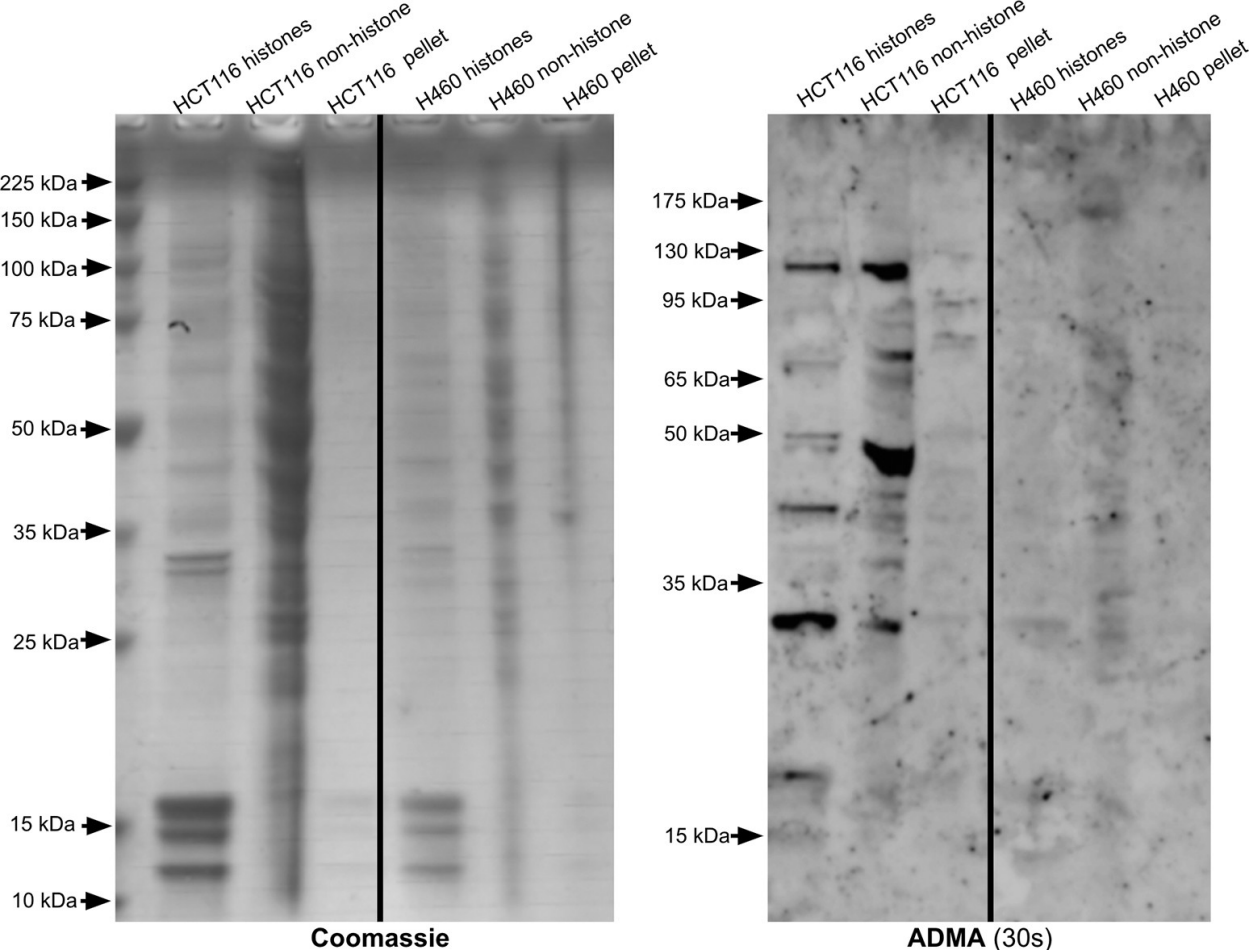
Identification of ADMA-containing polypeptides in histone and non-histone fractions of HCT116 and H460 cell extracts.

Because arginine methylation on histones can affect gene expression, we wanted to analyze any differences in SDMA and ADMA modifications on histones between each cell line. Upon histone enrichment in the HCT116 and H460 cells, a histone with SDMA with a molecular weight slightly above 15 kDa was observed (Fig 7 and S5 Fig). A similar result was seen in the 143B cells, where a non-histone 15 kDa protein and a histone protein slightly above 15 kDa both had the SDMA mark (Fig 3). A low signal for ADMA on a histone was seen in the HCT116 cells at slightly above 15 kDa (Fig 8). Meanwhile, the H460 cells do not appear to contain histones with ADMA, as there is little to no signal upon immunoblotting (Fig 8). The 143B histones also showed a signal near 15 kDa in the ADMA immunoblot (Fig 4). Overall, similarities in ADMA and SDMA on histones were observed between the HCT116 and 143B cells, while H460 cells seemed to lack ADMA on histones and non-histone proteins. These results support common patterns of protein arginine methylation in distinct cancer cell lines derived from various tissues. The significance of these common bands on anti-SDMA and anti-ADMA immunoblots is unknown and requires further experiments to elucidate their identity.

The left panel is a Coomassie-stained SDS-PAGE gel, and the right panel is an anti-SDMA immunoblot with an exposure time of 10 seconds. In this case, we utilized 12% SDS-PAGE gels (GenScript catalog #M01215). These images are representative of two individual experiments. Lanes from a single gel or blot were spliced together, as shown by the vertical black line, to remove irrelevant lanes.

The left panel is a Coomassie-stained SDS-PAGE gel, and the right panel is an anti-ADMA immunoblot with an exposure time of 30 seconds. In this case, we utilized 12% SDS-PAGE gels (GenScript catalog #M01215). These images are representative of two individual experiments. Lanes from a single gel or blot were spliced together, as shown by the vertical black line, to remove irrelevant lanes.

## Discussion

Whether there exists a link between methionine addiction and PRMT activity in cancer cells is still unknown, but the results presented here suggest that there is no large-scale direct connection in an osteosarcoma cell line. The rationale for initiating this study included the fact that methionine is the direct precursor of AdoMet, itself the substrate for PRMTs, as well as the known overexpression of PRMTs in many types of cancer cells [36–45]. Additionally, methionine-independent revertants of other cancer cell lines have greatly reduced transmethylation reactions compared to their parental cells [29, 30]. In the present study, we examined the levels of methylated proteins resulting from PRMT activity and substrate availability in methionine-independent revertant 143B human osteosarcoma cells and their parent cells to understand the mechanism behind the revertants’ loss of malignancy. Because the 143B-P cells relied more heavily on methionine than the 143B-R cells, different expression levels in protein arginine methylation were thought to be possible between parent and revertant cells. With the changes observed in markers of the epithelial-to-mesenchymal transition (EMT) and reported association between PRMTs and EMT, we expected there to be variations in proteins with ADMA and SDMA [28, 64]. Additionally, histone lysine methylation differed between the 143B-P and 143B-R cells, so we expected changes specifically in histone arginine methylation [28]. However, no major changes in protein arginine methylation were observed between the 143B-P and 143B-R cells, which suggests that changes in protein arginine methylation may not be central to the mechanism of reversion from methionine addiction to methionine independence.

In Fig 2, three proteins with SDMA with approximate molecular weights of 30 kDa, 20 kDa, and 15 kDa were seen at similar levels in the 143B-P and 143B-R cells. However, previous literature using a different SDMA antibody on an immunoblot of 143B cells also shows one major band near 85 kDa and a minor band near 128 kDa, which differs from the results presented here [65]. This may be due to differences in the antibody detection or differences in the method of cell culture. In both the 143B-P and 143B-R cells, multiple proteins with ADMA modifications are present between approximately 175 kDa and 30 kDa at similar intensity levels. Arginine methylation on histones can affect gene expression, so we hypothesized that SDMA and ADMA on histones may play a role in the mechanism of reversion. However, no major differences were observed in ADMA or SDMA on histones between the 143B-P and 143B-R cells (Figs 4 and 5).

While there appears to be some PRMT5 substrates that are methylated less in the revertant than the parent cell lysates, no major differences were found in substrate availability with an enzymatic assay using 143B-P and 143B-R cell lysates, [^3^H]-AdoMet, and PRMT enzymes (Fig 5). Because the banding pattern is similar between reactions with lysates only and those with PRMT enzymes added, it is possible that these bands show other types of protein methylation that are not catalyzed by PRMTs. While only arginine methylation was directly tested here, there appears to be no major differences in cytoplasmic protein methylation as a whole because the 143B-P and 143B-R cell lysates without PRMTs and with [^3^H]-AdoMet show the same radioactive banding pattern. Although changes in histone lysine methylation between the 143B-P and 143B-R cells were seen previously, the method of cell lysis may not have lysed the nuclear membrane and did not solubilize histones, as shown in the Coomassie-stained gel that lacks histone bands [28]. Other than in protein methylation, AdoMet is the methyl donor in DNA and RNA methylation, which both can be deregulated in cancer [60, 66]. Twenhafel et. al has reviewed how epigenetic changes are associated with disease progression in osteosarcoma [67]. DNA methylation can suppress miRNAs, whose underexpression in osteosarcoma tissue is correlated with higher-stage tumors. Additionally, DNA methylation of tumor-suppressing genes can silence their transcription and therefore allow for further cancer progression. Methylation of RNA at adenosine bases leads to N6-methyladenosine (m6a), which is recognized by “reader” proteins, promoting translation of the mRNA. In osteosarcoma, methyltransferase-like protein 3, which adds a methyl group to form m6a, is linked to tumor progression. A methionine-restricted diet in a mouse model of colorectal carcinoma was shown to decrease m6a mRNA methylation in cancer cells [68]. DNA and RNA methylation were not explored in this study, but they could be altered as a result of a lower dependence on methionine. Because methionine metabolism includes pathways besides protein methylation, there are many possibilities for the mechanism of reversion from methionine addiction to methionine independence. These results suggest that protein arginine methylation may not be important in altering the osteosarcoma cells’ methionine addiction. Interestingly, a previous study has shown that PRMT1 and methionine are both required for Wnt signaling activity in HeLa cells [69]. Methionine deprivation decreased PRMT1 colocalization with glycogen synthase kinase 3, an essential enzyme in Wnt signaling [69]. Thus, the association between methionine addiction and PRMT function may vary among different types of cancer cells.

PRMTs are generally overexpressed in cancer, and this study showed a direct comparison of proteins with SDMA and ADMA within various cancer cell lines. In the 143B, HCT116, and HeLa cells, many proteins with ADMA are present upon immunoblotting across a wide range of molecular weights (Fig 6). In previous literature, multiple bands ranging from 25 kDa to 150 kDa are also shown in an anti-ADMA immunoblot of an HCT116 cell lysate [70]. Additionally, human embryonic kidney 293T cells have multiple proteins with ADMA between 20kDa and 250 kDa, as well as three SDMA-containing proteins near 15 kDa, 20 kDa, and 30 kDa [57]. A previous report of ADMA in breast tumors found 403 ADMA sites on 213 proteins in patient-derived xenograft tumors, which highlights the extensive methylation by Type I PRMTs in cancerous tissues [43]. The HCT116 and HeLa cells had the highest abundance of proteins with SDMA. HCT116 cells express the methylthioadenosine phosphorylase (MTAP) protein at a higher level than H460 cells, while 143B cells do not express MTAP [29, 71]. HeLa cells do express MTAP [72]. Without the MTAP enzyme, methylthioadenosine can accumulate since it cannot be phosphorylated by MTAP [54, 73]. Methylthioadenosine can inhibit PRMT5, which is the enzyme responsible for most SDMA in the cell [73]. This endogenous inhibition of PRMT5 mediated by *MTAP* deletion may affect SDMA levels because there are only three major bands on the SDMA immunoblot of the 143B cells. The relatively low abundance of proteins with SDMA in the 143B cells may be due to *MTAP* deletion. *MTAP*-deleted cell lines are more sensitive to methioninase treatment than those containing the *MTAP* gene, so it is surprising that the 143B-P and 143B-R cells did not have any major differences in PRMT5 activity [71]. Because they are more sensitive to PRMT inhibition, *MTAP*-deleted cancers can be selectively targeted [73]. Identifying the major substrates of PRMT5 that are still methylated in *MTAP*-deleted cancers may be important to understanding what reactions PRMT inhibitors are acting on in these cancers. Further experiments are necessary to elucidate the identity and importance of the common proteins with SDMA and ADMA found in the several cancer cell lines studied here.

While we have shown that PRMT activity may not be connected to methionine addiction as well as similarities in PRMT substrates between cancer cell lines, which are also methionine-addicted [29, 74], there are a few limitations to this study. Methionine-addicted cancer cell lines grown in methionine-depleted medium have low levels of free intracellular methionine as shown in Table 1 of Stern et. al, 1983 [8]. Whether there are differences in methionine concentrations between the 143B-P and 143-R cells remains unknown; however, it appears that any potential differences did not affect protein arginine methylation. Also, protein arginine methylation is stable in cells [75, 76]. HeLa cell extracts treated with adenosine dialdehyde, a methyltransferase inhibitor, still had many proteins with SDMA and ADMA present, although at a slightly lower level than HeLa cells cultured without any inhibitor [75]. Using [^3^H]-AdoMet, recombinantly purified PRMTs, and cell lysates to identify substrates may not account for stable proteins that have been modified by endogenous PRMTs during cell culture. Analyzing PRMT activity with similar methods but in synchronized cells would clarify whether variations exist in protein arginine methylation at different points in the cell cycle. PRMT3, 6, and 7 are differentially expressed depending on the stage of the cell cycle in synchronized HeLa cells [77]. While no differences in protein arginine methylation between the 143B-P and 143B-R cells were observed, it is possible that changes in ADMA may occur at specific points during cell growth and be observed in synchronized cells. Because overexpression of the PRMT enzymes is common in cancer cells, measuring the expression levels of each PRMT within the 143B-P and 143B-R cells would be an important future study to further elucidate the mechanisms of methionine addiction. Additionally, PRMT substrates may be different in vivo. A study analyzing ADMA on proteins in ER- and ER+ breast cancer showed a low amount of shared ADMA sites between tumor samples and in vitro samples [43]. While this study compared the abundance of SDMA and ADMA on proteins between various human cancer cell lines, a similar comparison among primary tumors in different tissues may produce distinct results. Finally, immunohistochemistry analyses could determine the localization of SDMA and ADMA containing proteins within parent and revertant cells. While this study has suggested no association between methionine addiction and protein arginine methylation in osteosarcoma, the mechanism of methionine addiction remains unknown. Future studies could use proteomics and RNAseq analyses in parent and revertant cells to elucidate the pathways important for methionine dependency.

Overall, elucidating the mechanism in which methionine restriction can affect cancer malignancy will be useful in developing targeted cancer therapeutics. With PRMT inhibitors entering clinical trials for cancer patients, it is important to understand how methionine deprivation on its own and in combination with these drugs may affect PRMT activity. The present results suggest that methionine addiction and the upregulation of PRMTs are distinct characteristics of osteosarcoma cells because the revertant cells with lowered methionine dependence showed no changes in PRMT activity.

## Materials and methods

### Preparation of cells and other materials

The 143B parent and revertant cell lines were cultured as previously described [28]. The HCT116 and H460 cells used in this study were the parent cell lines previously described in Yamamoto et. al [29]. Approximately 2,000,000 cells each of the HCT116, H460, 143B-P, and 143B-R grown in high-methionine medium were shipped to UCLA as a cell pellet on ice at 0°C from AntiCancer, Inc. A frozen cell pellet of approximately 500,000,000 cells of the HeLa-S3 epithelial suspension adapted cell line (C3 PN:HA48) was purchased from the Cell Culture Company (Minneapolis, MN). Upon arrival, the cells were either frozen at -80°C or were kept at 0°C. Cells were aliquoted by suspending them in 1 mL of 1X PBS (Phosphate Buffered Saline 10X Solution, Fisher Scientific, catalog #BP3991, lot #124561) and divided into approximately 250,000-cell aliquots for the HCT116, H460, and HeLa cells, and 200,000-cell aliquots for the 143B-P and 143B-R cells. In each case, the cells were spun down at 180 *g* for 5 min at 4°C. The new cell pellets and their PBS supernatants were stored separately at -80°C for future use. Uninduced, untransformed BL21 *E*. *coli* were cultured in 10 mL LB media overnight at 37°C, leaving a final OD600 of 1.29. The cells were divided into 500 μL aliquots, which were lysed at 13,000 *g* at 4°C for 5 min.

### Histone extraction

The EpiQuick Total Histone Extraction Kit (EpiGentek, catalog #OP-0006) was used to extract the histones from each cell line. One aliquot (250,000 cells for H460 and HCT116, and 200,000 cells for 143B-P and 143B-R cells) was used for each histone extraction, which resulted in 26 μL of histone material. The supernatant with non-histone proteins and the unextracted pellet that remained after using the kit were saved, and their proteins were fractionated on SDS-PAGE along with the histone-containing supernatants. For SDS-PAGE, the volume of sample applied was normalized to the protein concentration of histones measured using 3 μL of the histone fraction on a NanoDrop 2000c spectrophotometer. The absorption at 280 nm was calculated, where 1 mg/mL was assumed to give 1 A.

### SDS-PAGE and immunoblotting

Each 200,000-cell 143B pellet, 250,000-cell H460 pellet, 250,000-cell HCT116 pellet, or 250,000-cell HeLa pellet was suspended in 200 μL 2X sample buffer (4% SDS, 20% glycerol, 120 mM Tris-HCl pH 6.8, 0.02% bromophenol blue, 5% beta-mercaptoethanol). Histone extraction fractions were diluted in 2X sample buffer or 5X sample buffer (10% SDS, 50% glycerol, 250 mM Tris-HCl pH 6.8, 0.05% bromophenol blue, 12.5% beta-mercaptoethanol). A 1.3 mg/mL solution of GST-GAR, prepared as described in Lowe et. al [62], was diluted in 2X sample buffer, and 6.5 μg were loaded onto the gel. Each *E*. *coli* pellet was resuspended in 130 μL 2X sample buffer, and 15 μL were loaded onto the gel. For the purified histones, samples were analyzed of the histone fraction, the non-histone soluble fraction, and the non-histone pellets obtained from the histone extraction kit described above. In Figs 3 and 4, histones and non-histone supernatants were diluted in 5X sample buffer at a 1:4 ratio, and non-histone pellets were resuspended in 50 μL 5X sample buffer. 15 μL of the non-histone supernatant and non-histone pellet were loaded into each well. For Fig 3, the 143B-P and 143B-R histones contained 1.6 mg/mL and 2.1 mg/mL, respectively, so 15 μL 143B-P histones and 11 μL 143B-R histones (19 μg) were loaded onto the gel. For Fig 4, the 143B-P and 143B-R histones contained 1.2 mg/mL and 0.7 mg/mL, respectively, so 11 μL 143B-P histones and 20 μL 143B-R histones (11 μg) were loaded onto the gel. In Figs 7 and 8, histones and non-histone supernatants were diluted in 2X sample buffer at a 1:1 ratio, and non-histone pellets were resuspended in 200 μL 2X sample buffer. 15 μL of the non-histone supernatant and non-histone pellet were loaded into each well. For Fig 7, the HCT116 and H460 histones contained 5.8 mg/mL and 2.3 mg/mL, respectively, so 8 μL HCT116 histones and 20 μL H460 histones (23 μg) were loaded onto the gel. For Fig 8, the HCT116 and H460 histones contains 2.2 mg/mL and 3.9 mg/mL, respectively, so 15 μL HCT116 histones and 9 μL H460 histones (17 μg) were loaded onto the gel. Whole cell extracts in 2X sample buffer were drawn up and down 20 times in a 1 mL syringe with luer-lock and a 20 gauge needle (BD PrecisionGlide™ Needle; 20G * 1 ½ (0.9mm * 40mm)) to shear the DNA. Each sample was heated at 95°C for 3 minutes. The whole cell extract was separated with 35 μL in each well on a 4–20% (unless otherwise mentioned) ExpressPlus PAGE gel (GenScript catalog #M42010) at 140V for 1 hour using a BioRad PowerPac 300 electrophoresis Power supply with Tris-MOPS-SDS running buffer (GenScript Tris-MOPS-SDS Running Buffer Powder; 50 mM Tris Base, 50 mM MOPS, 0.1% SDS, 1 mM EDTA). Coomassie staining was performed on a separate gel with identical loading as a control for the amount of protein. Gels were stained for 1 hour with Coomassie (0.1% (w/v) Brilliant Blue R-250, 10% (v/v) glacial acetic acid, and 50% (v/v) methanol) and destained overnight with 10% (v/v) acetic acid and 15% (v/v) methanol. For immunoblotting, gels were transferred onto a PVDF membrane with transfer buffer (10% (v/v) Tris-glycine, 10% (v/v) methanol) at 30V for 2 hours with ice using a BioRad PowerPac 300 electrophoresis Power supply. The blocking buffer used was 5% bovine serum albumin (BSA) in TBS-T (20 mM Tris, 137 mM NaCl, 0.1% (v/v) Tween-20, pH 7.6). The primary antibodies used in this study were rabbit polyclonal anti-SDMA, anti-ADMA, and anti-MMA (1:2000, generously gifted from Dr. Mark T. Bedford [57]). The immunogens were short GAR motifs with either the SDMA, ADMA, or MMA modification separated by polyethylene glycol linkers. The secondary antibody used was goat anti-rabbit IgG (H + L) Alexa Fluor Plus 647 (1:2000, Thermo Fisher, catalog #A32733). All antibody solutions were diluted in 5% BSA in TBS-T. Molecular weights of proteins were calculated using ImageJ (Version 2.1.0/1.53c).

### SDS-PAGE fluorography with [^3^H]AdoMet

Approximately 1,000,000 cells were lysed in 100 μL of NP-40 cell lysis buffer (1 EDTA-free protease inhibitor tablet per 50 mL (Catalog No. PIA32965, Thermo Scientific), 50 mM HEPES, pH 7.6, 150 mM NaCl, 7% beta-mercaptoethanol, 1% NP-40). Cells were lysed at 13,000 *g* for 1 minute. Each reaction mixture contained 33.3% (v/v) cell lysate, 50 mM HEPES pH = 9.0 (9.5 for PRMT7), 1 mM DTT, and 0.14 μM *S*-adenosyl-ʟ-[methyl-^3^H] methionine ([^3^H]AdoMet; Catalog No. NET155H001MC, PerkinElmer Life Sciences; 81.9 Ci/mmol, 7 μM in 9:1 10 mM H_2_SO_4_:ethanol). Reactions with PRMTs contained either 0.20 μg PRMT1, 0.076 μg PRMT5, or 5.5 μg PRMT7. Each reaction also contained 2 μM MS023 (MedChemExpress, catalog # HY-19615) [78], 20 μM EPZ015666 (MedChemExpress, catalog # HY-12727) [79], 200 nM SGC8158 [80], or 3.33% (v/v) DMSO. Incubation temperature was 37°C for PRMT1, PRMT5, and controls, and 20°C for PRMT7. Incubation time was one hour. Reactions were terminated with 5X sample buffer, and 30 μL of the reaction were fractionated with SDS-PAGE as described above. Gels were incubated with EN^3^HANCE (PerkinElmer Life Sciences, catalog #6NE9701) for 1 hour, incubated in water for 30 minutes, and dried before the gels were exposed to film (Denville Scientific, 8 X 10 in. Hyblot Cl) at -80°C for 63 days. The eight reactions consisted of the following: lysate only; PRMT1, lysate; PRMT5, lysate; PRMT7, lysate; PRMT1, lysate, EPZ015666, SGC8158; PRMT5, lysate, MS023, SGC8158; PRMT7, lysate, MS023, EPZ015666; lysate, no enzyme, MS023, EPZ015666, SGC8158.

## Supporting information

S1 Fig Validating antibodies against SDMA and ADMA. 143B-R whole cell extracts were used as positive controls, and GST-GAR and *E*. *coli* extracts were used as negative controls. To ensure the antibodies recognized the arginine methylation modification rather than the glycine and arginine rich regions, an unmethylated GST-tagged protein, based on the N-terminus of human fibrillarin that contains a glycine and arginine rich region (GST-GAR), was used as a negative control. Another negative control was an extract of untransformed and uninduced BL21 *Escherichia coli* (*E*. *coli*) lysate. The first and third panels show Coomassie-stained SDS-PAGE gels. The second and fourth panels are immunoblots using the SDMA antibody or ADMA antibody with exposure times of 3 seconds or 10 seconds, respectively. Lanes from a single gel or blot were spliced together, as shown by the vertical black line, to remove irrelevant lanes. The same lanes for the parent cells and parent histones are shown in Figs 2 and 4, respectively.
(TIF)

S2 Fig Identification of proteins containing SDMA and ADMA in human parent and revertant 143B osteosarcoma cells: Replicate experiment. This shows an individual replicate experiment of the same conditions as in Fig 2. The leftmost panel shows a Coomassie-stained SDS-PAGE gel. The middle panel and rightmost panel show an anti-SDMA immunoblot and an anti-ADMA immunoblot, respectively, each with a 30 second exposure time. Molecular weight markers are shown at the left in the Coomassie-stained gel and as fluorescent bands on the left margin of the immunoblots.
(TIF)

S3 Fig Identification of SDMA-containing polypeptides in histone and non-histone fractions of parent and revertant human osteosarcoma 143B cells. This shows a higher exposure (30 seconds) of the immunoblot in Fig 3 to show the histone band more clearly.
(TIF)

S4 Fig Identification of proteins containing SDMA and ADMA in whole cell extracts of the human colorectal cancer HCT116 cell line, human cervical cancer HeLa cell line, and human non-small cell lung cancer H460 cell line. This shows a higher exposure (10 seconds) of the immunoblots in Fig 6. The left panel is an anti-SDMA immunoblot, and the right panel is an anti-ADMA immunoblot.
(TIF)

S5 Fig Identification of SDMA-containing polypeptides in histone and non-histone fractions of HCT116 and H460 cell extracts. This shows a higher exposure (30 seconds) of the anti-SDMA immunoblot in Fig 7.
(TIF)

S1 Raw images Original data for all immunoblots. (PDF)

S1 File Original data for Fig 1 graph. (XLSX)
