# Supplementary figures and images for: Asymmetric and symmetric protein arginine methylation in methionine-addicted human cancer cells
Source: PLoS One. 2023 Dec 22;18(12):e0296291. doi: 10.1371/journal.pone.0296291 (PMC10745221; doi:10.1371/journal.pone.0296291)

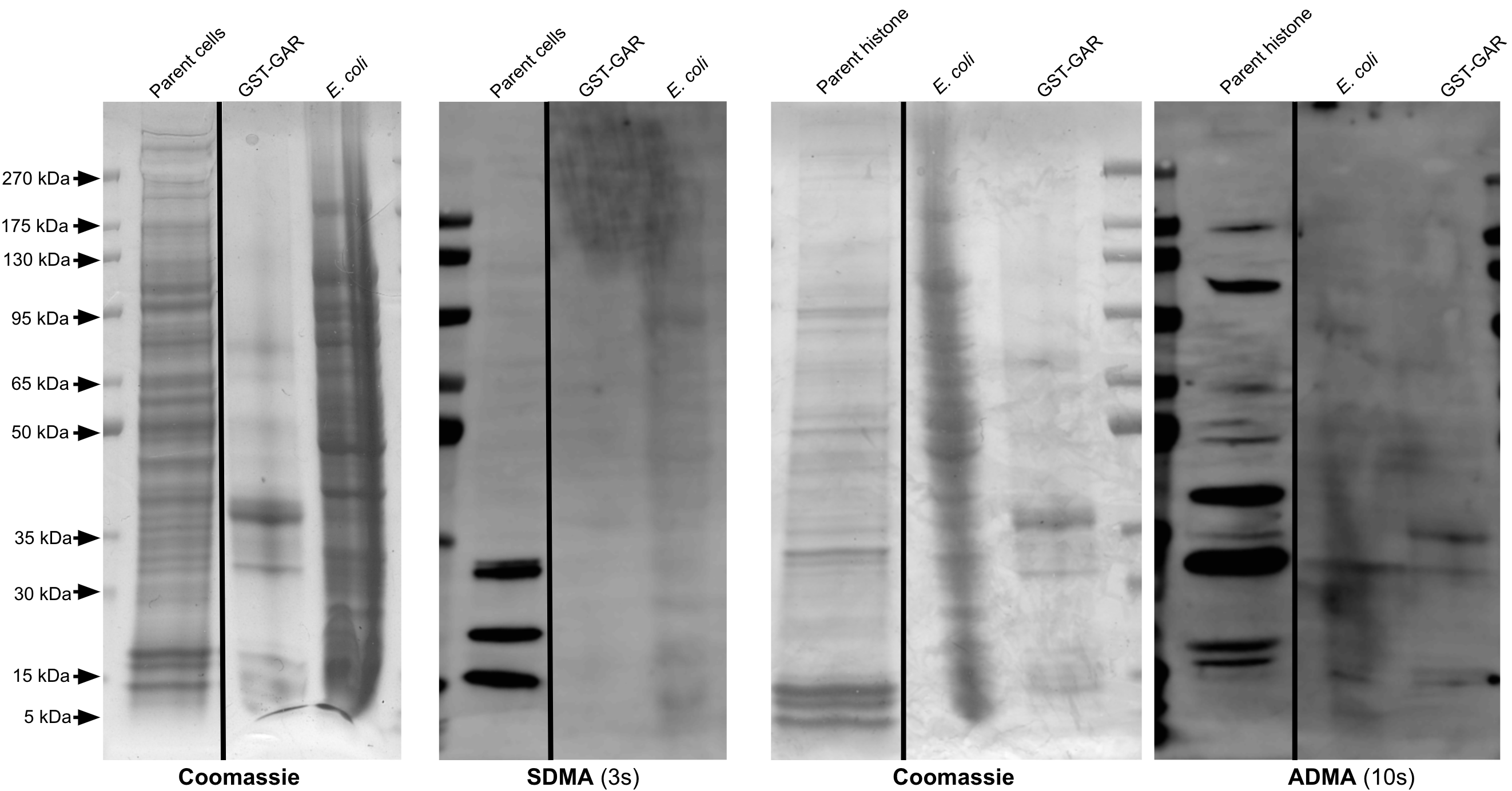

Supplement: S1 Fig — 143B-R whole cell extracts were used as positive controls, and GST-GAR and E. coli extracts were used as negative controls. To ensure the antibodies recognized the arginine methylation modification rather than the glycine and arginine rich regions, an unmethylated GST-tagged protein, based on the N-terminus of human fibrillarin that contains a glycine and arginine rich region (GST-GAR), was used as a negative control. Another negative control was an extract of untransformed and uninduced BL21 Escherichia coli (E. coli) lysate. The first and third panels show Coomassie-stained SDS-PAGE gels. The second and fourth panels are immunoblots using the SDMA antibody or ADMA antibody with exposure times of 3 seconds or 10 seconds, respectively. Lanes from a single gel or blot were spliced together, as shown by the vertical black line, to remove irrelevant lanes. The same lanes for the parent cells and parent histones are shown in Figs 2 and 4, respectively. (TIF) [file pone.0296291.s001.tif]

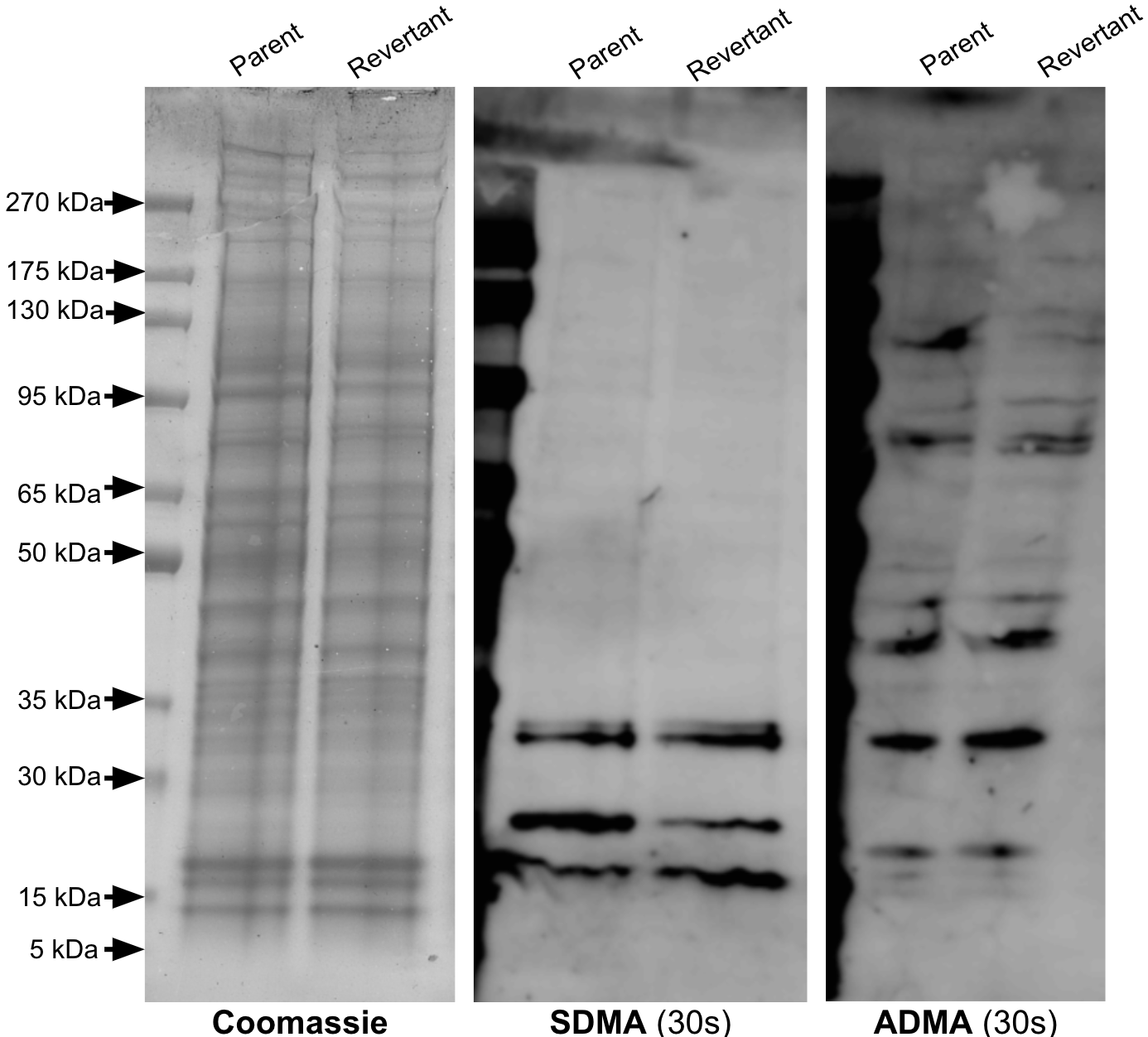

Supplement: S2 Fig — This shows an individual replicate experiment of the same conditions as in Fig 2. The leftmost panel shows a Coomassie-stained SDS-PAGE gel. The middle panel and rightmost panel show an anti-SDMA immunoblot and an anti-ADMA immunoblot, respectively, each with a 30 second exposure time. Molecular weight markers are shown at the left in the Coomassie-stained gel and as fluorescent bands on the left margin of the immunoblots. (TIF) [file pone.0296291.s002.tif]

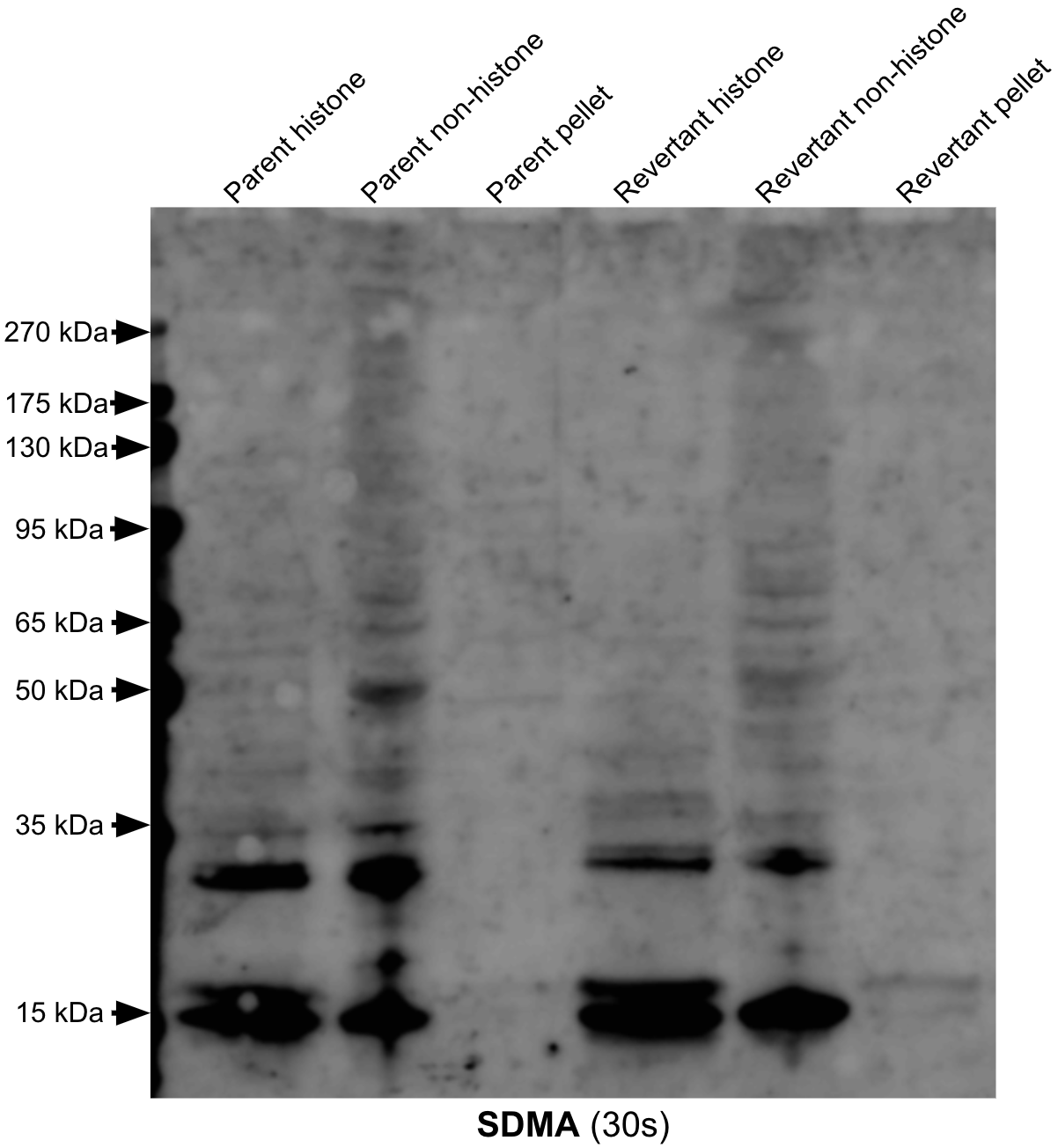

Supplement: S3 Fig — This shows a higher exposure (30 seconds) of the immunoblot in Fig 3 to show the histone band more clearly. (TIF) [file pone.0296291.s003.tif]

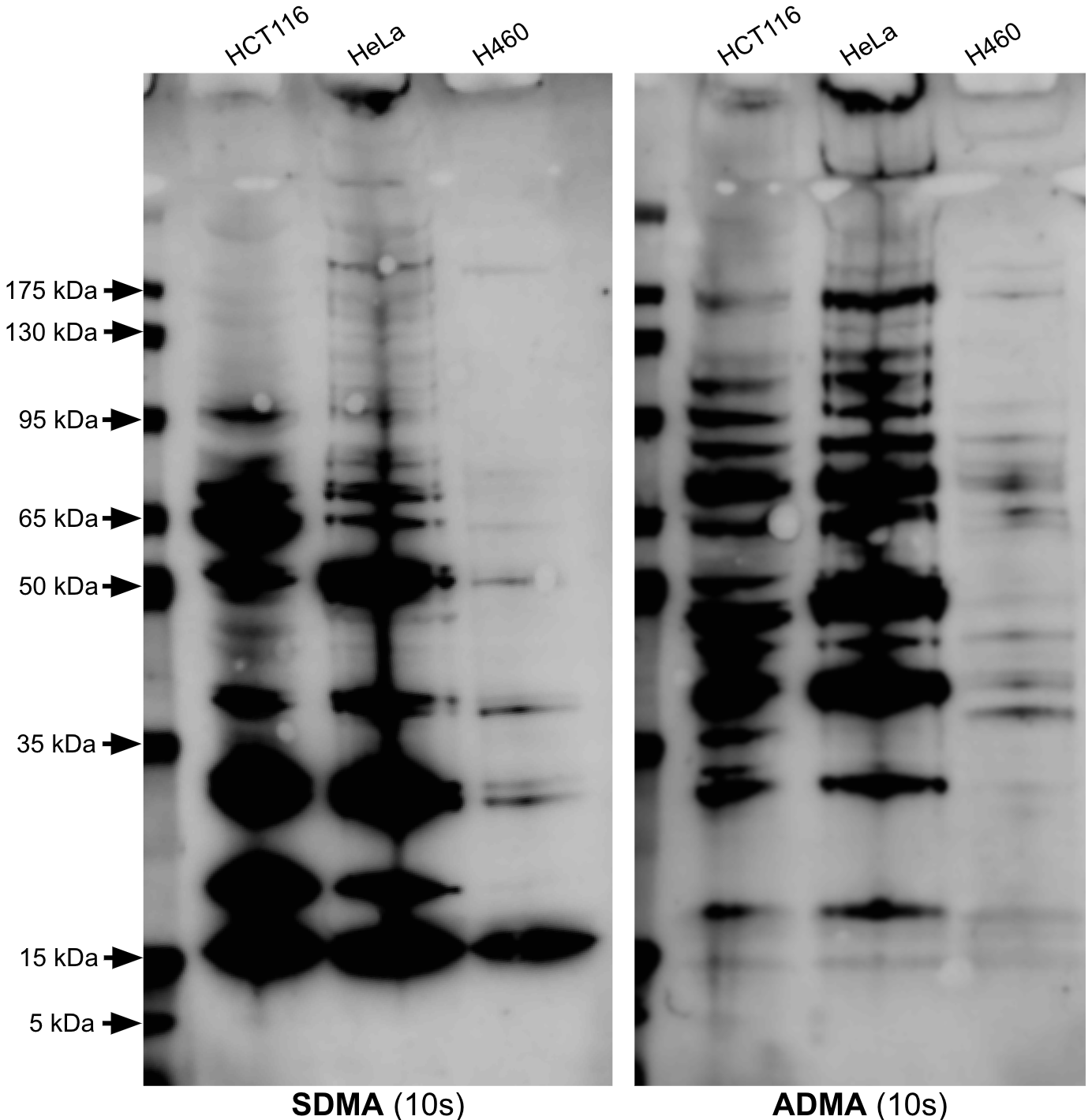

Supplement: S4 Fig — This shows a higher exposure (10 seconds) of the immunoblots in Fig 6. The left panel is an anti-SDMA immunoblot, and the right panel is an anti-ADMA immunoblot. (TIF) [file pone.0296291.s004.tif]

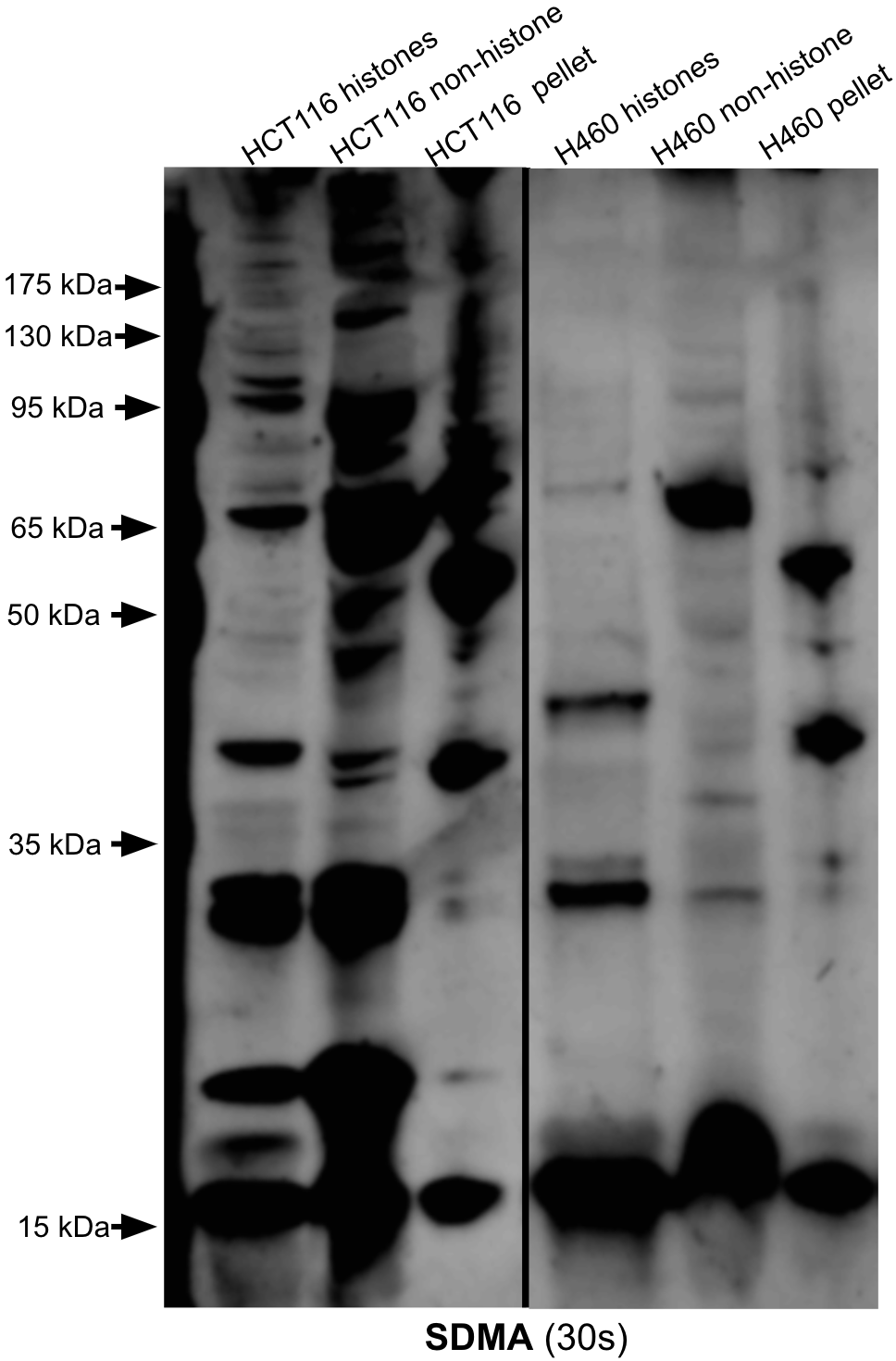

Supplement: S5 Fig — This shows a higher exposure (30 seconds) of the anti-SDMA immunoblot in Fig 7. (TIF) [file pone.0296291.s005.tif]
