# Supplementary material for: Asymmetric and symmetric protein arginine methylation in methionine-addicted human cancer cells
Source: PLoS One. 2023 Dec 22;18(12):e0296291. doi: 10.1371/journal.pone.0296291 (PMC10745221; doi:10.1371/journal.pone.0296291)
Supplement: S1 Raw images — (PDF) [file pone.0296291.s006.pdf]

Figure S1 and Figure 2:

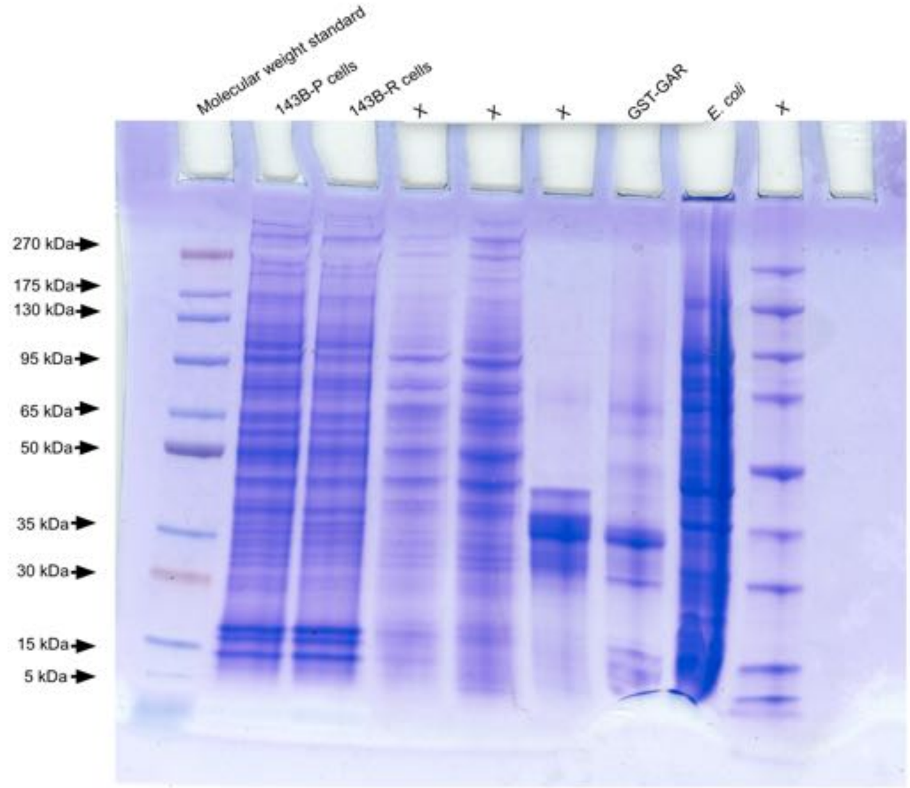

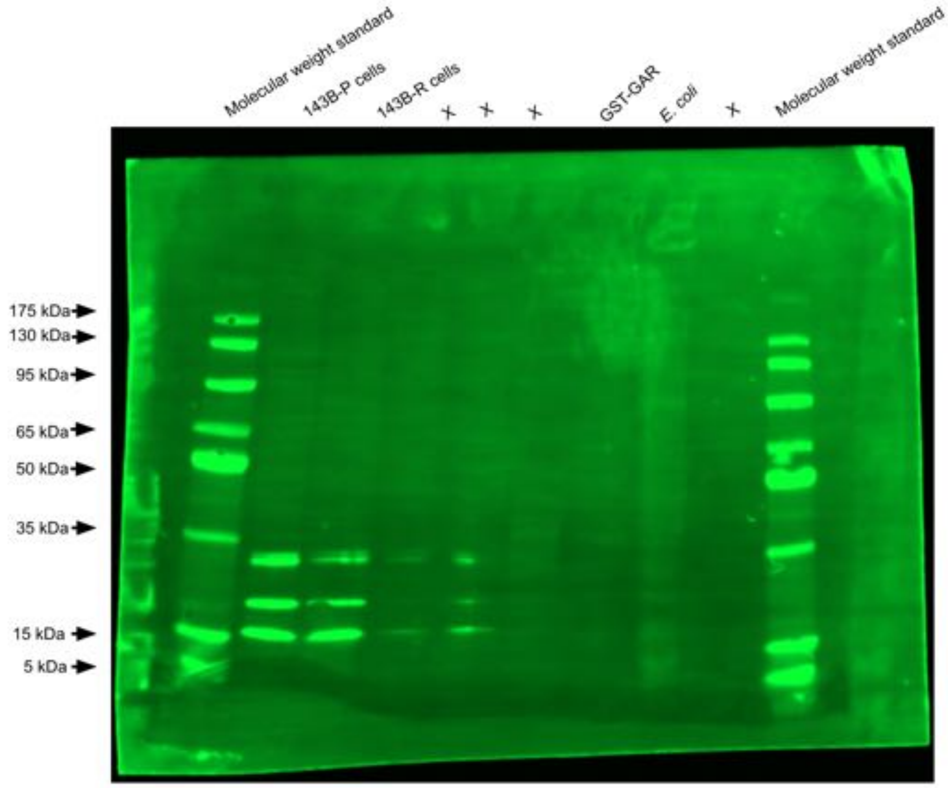

Figure 3:

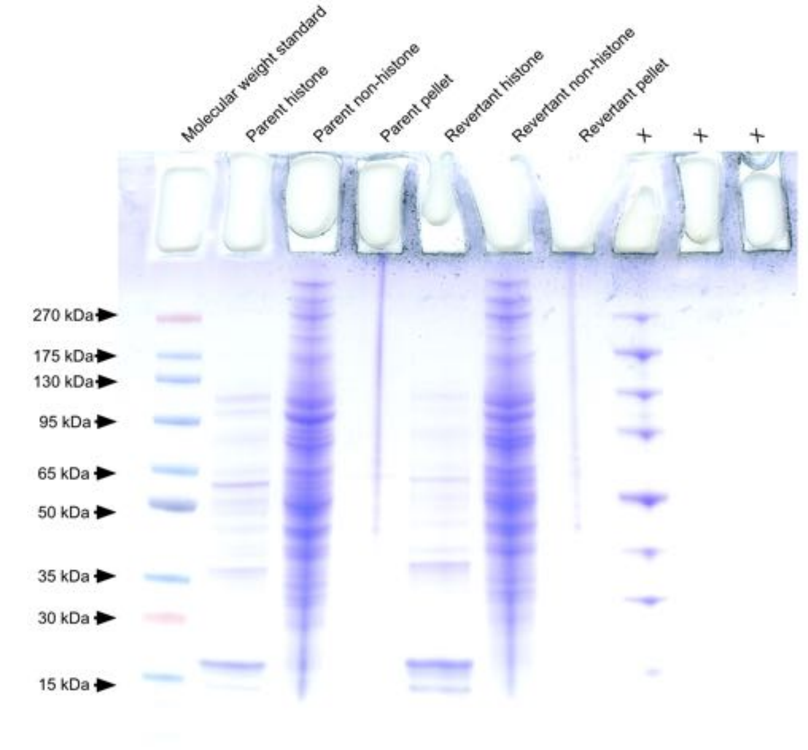
Molecular weight standard
Parent histone
Parent non-histone
Parent pellet
Revertant histone
Revertant non-histone
Revertant pellet
X
X
X
270 kDa
175 kDa
130 kDa
95 kDa
65 kDa
50 kDa
35 kDa
30 kDa
15 kDa

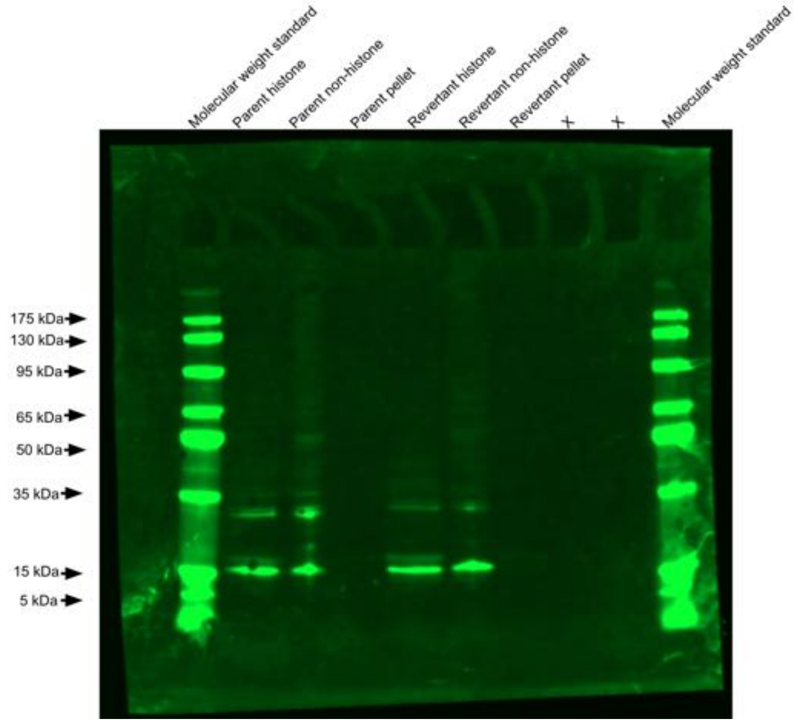
Molecular weight standard
Parent histone
Parent non-histone
Parent pellet
Revertant histone
Revertant non-histone
Revertant pellet
X
X
Molecular weight standard
175 kDa
130 kDa
95 kDa
65 kDa
50 kDa
35 kDa
15 kDa
5 kDa

Figure S1 and Figure 4:

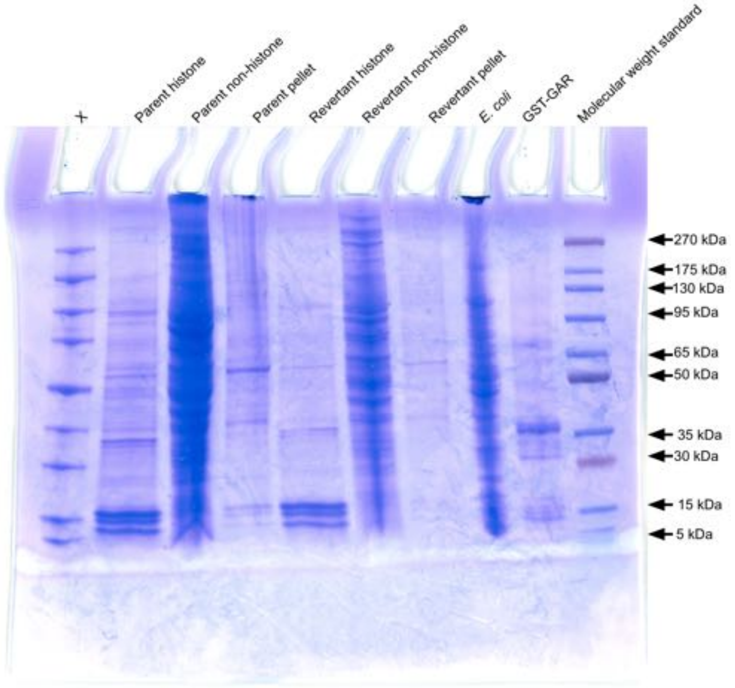

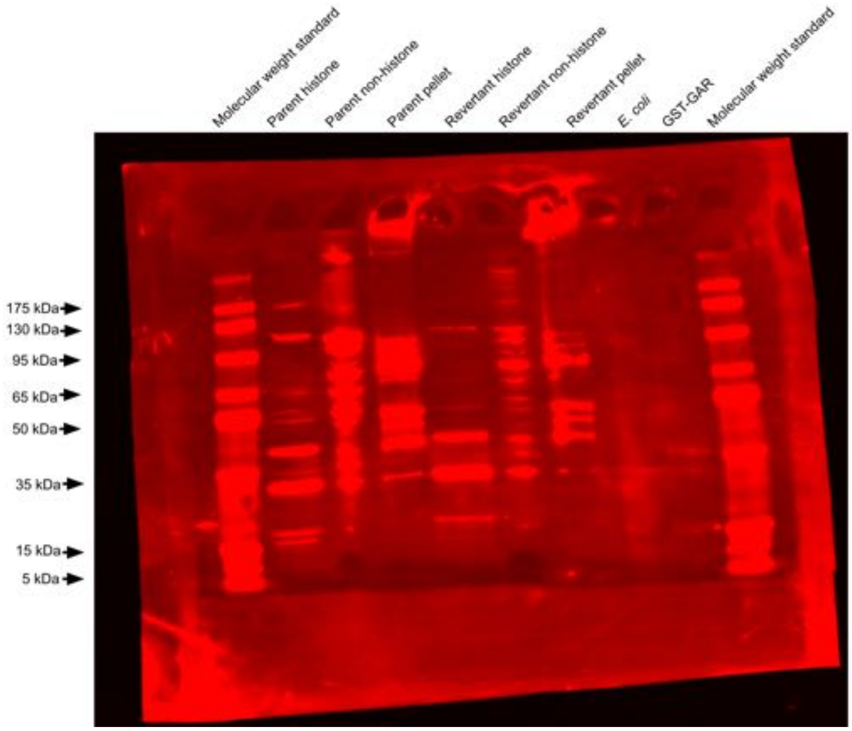

Figure 5:

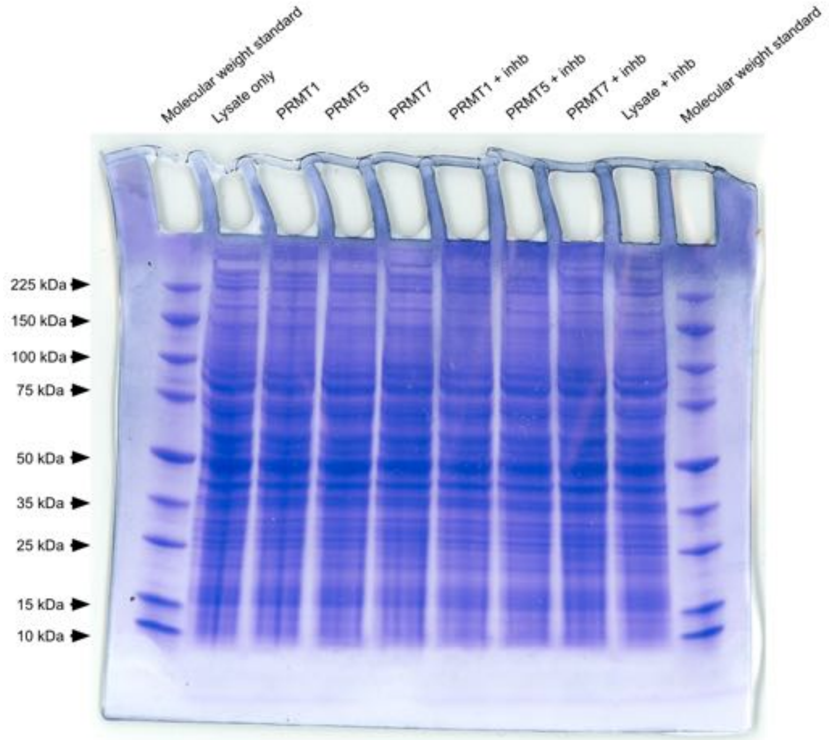
Molecular weight standard
Lysate only
PRMT1
PRMT5
PRMT7
PRMT1 + inhb
PRMT5 + inhb
PRMT7 + inhb
Lysate + inhb
Molecular weight standard
225 kDa
150 kDa
100 kDa
75 kDa
50 kDa
35 kDa
25 kDa
15 kDa
10 kDa

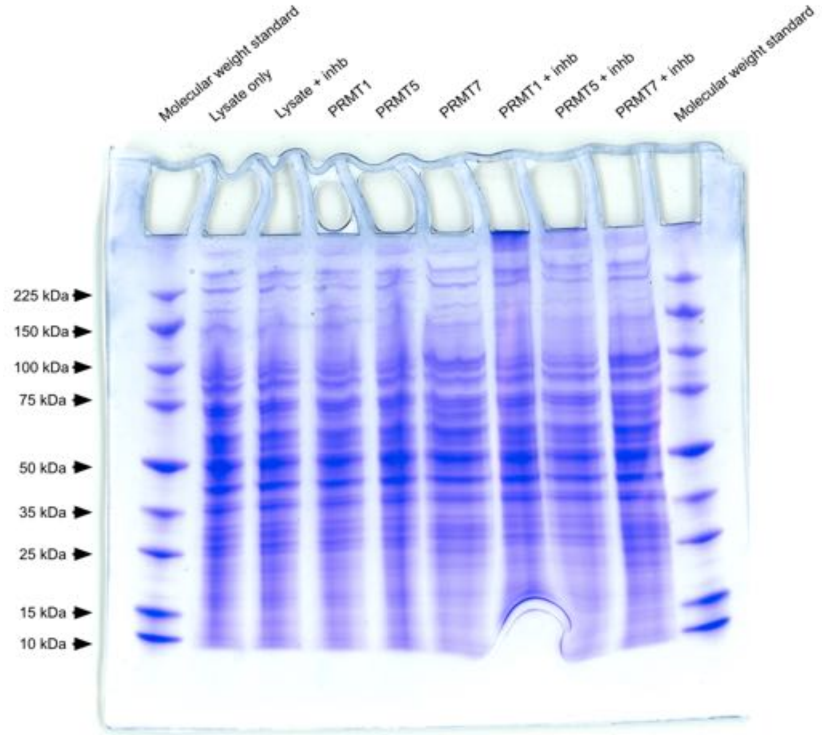
Molecular weight standard
Lysate only
Lysate + inhb
PRMT1
PRMT5
PRMT7
PRMT1 + inhb
PRMT5 + inhb
PRMT7 + inhb
Molecular weight standard
225 kDa
150 kDa
100 kDa
75 kDa
50 kDa
35 kDa
25 kDa
15 kDa
10 kDa

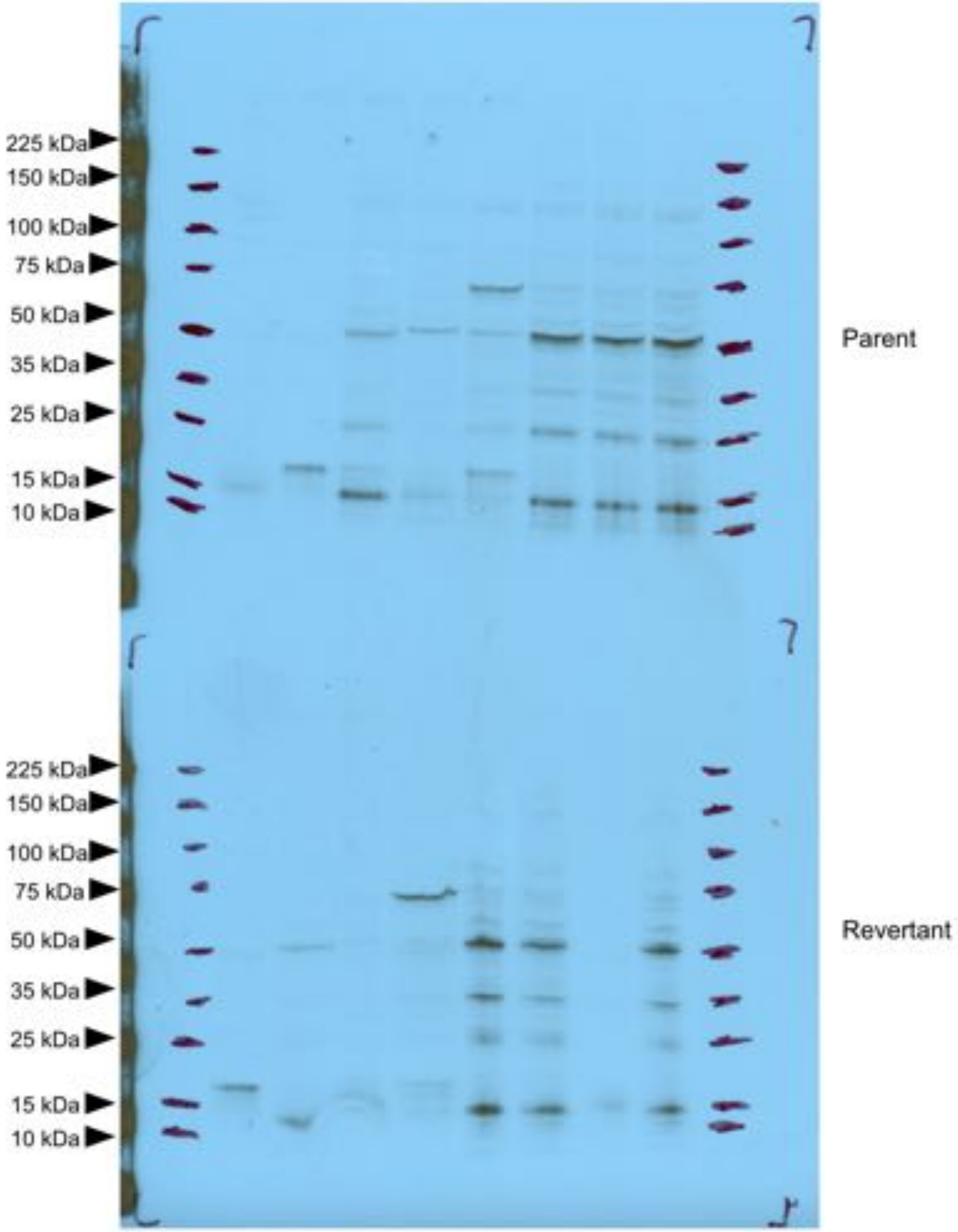
225 kDa
150 kDa
100 kDa
75 kDa
50 kDa
35 kDa
25 kDa
15 kDa
10 kDa
Parent
225 kDa
150 kDa
100 kDa
75 kDa
50 kDa
35 kDa
25 kDa
15 kDa
10 kDa
Revertant

Figure 6:

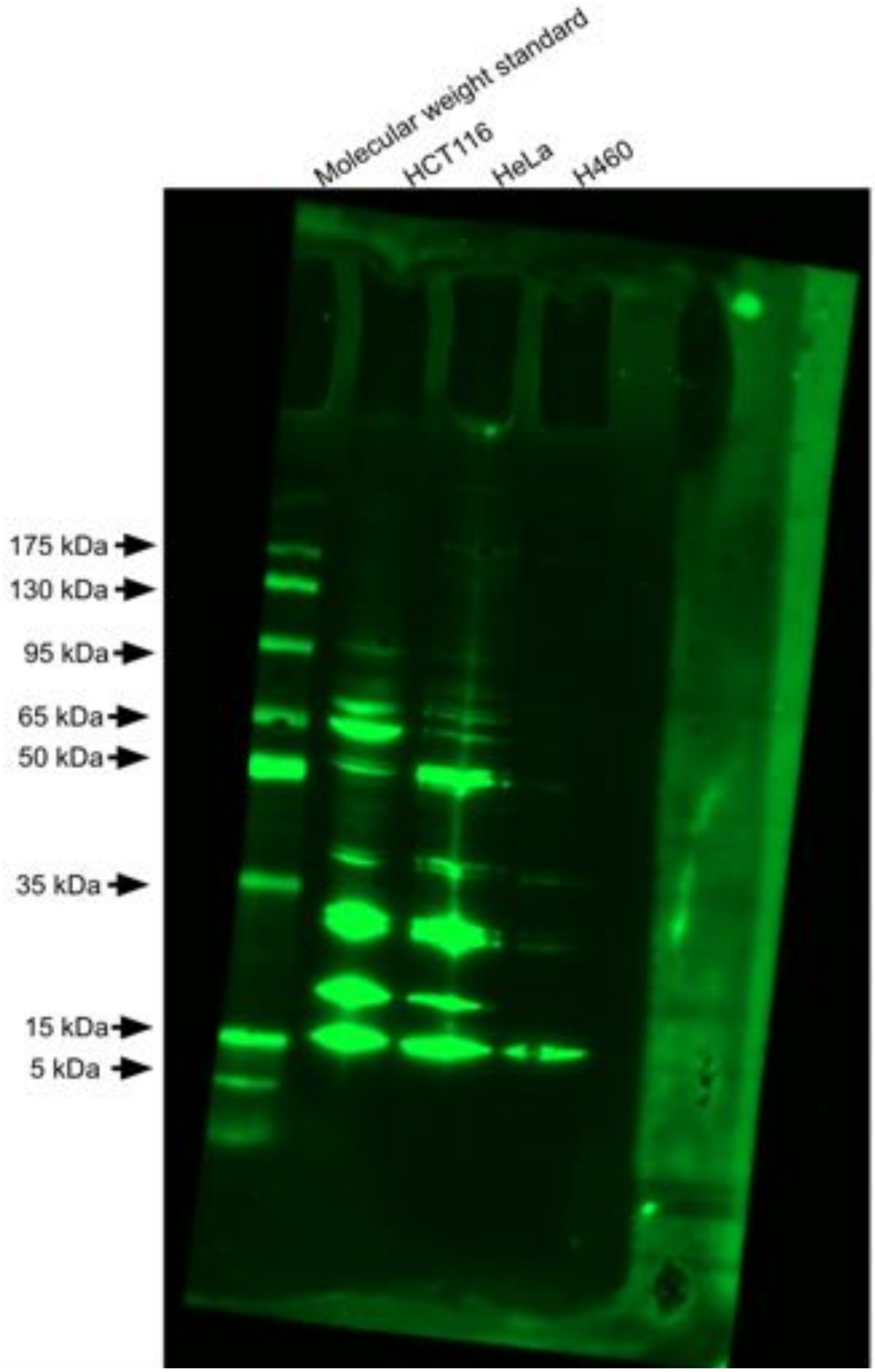
Molecular weight standard
HCT116
HeLa
H460
175 kDa
130 kDa
95 kDa
65 kDa
50 kDa
35 kDa
15 kDa
5 kDa

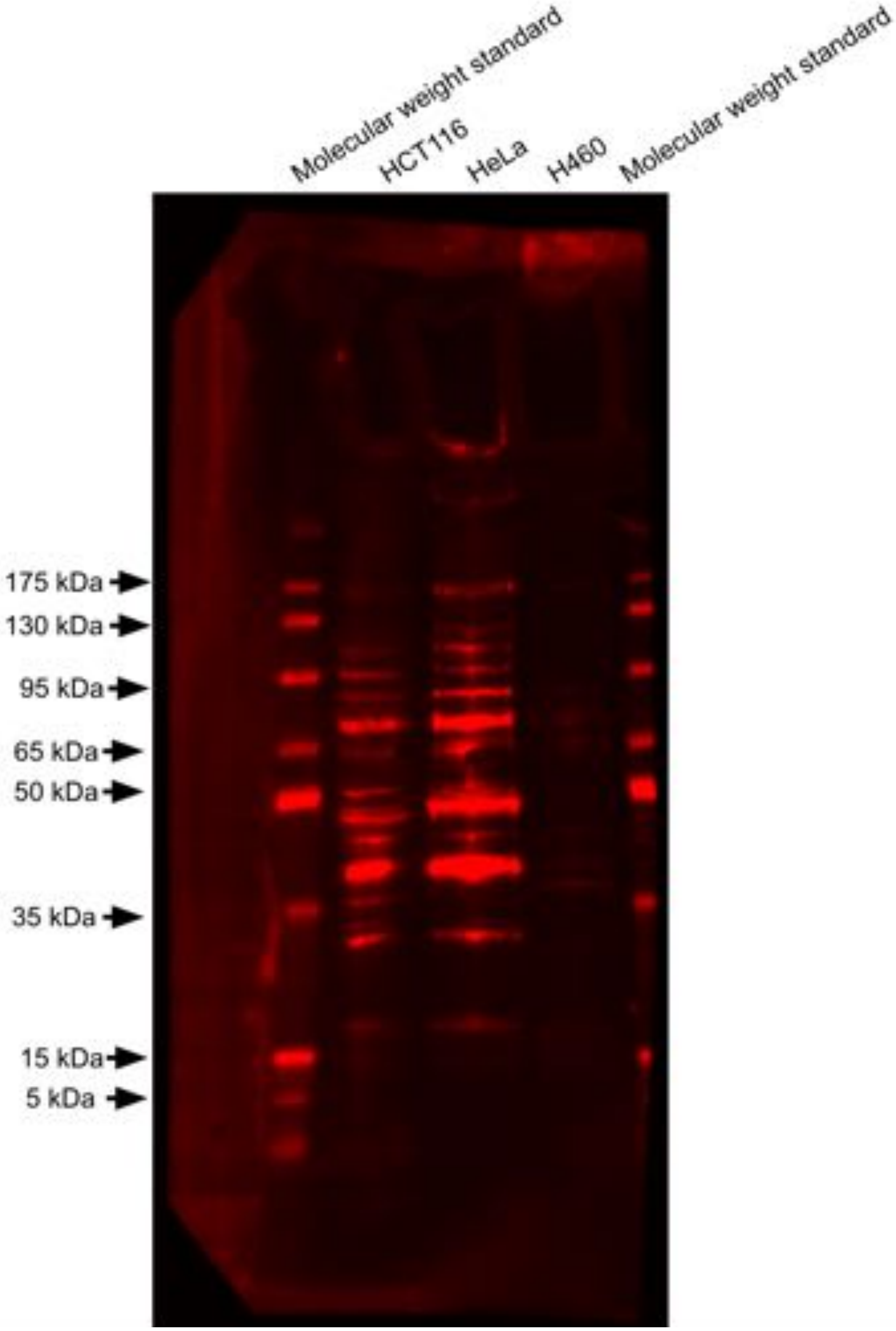
Molecular weight standard
HCT116
HeLa
H460
Molecular weight standard
175 kDa
130 kDa
95 kDa
65 kDa
50 kDa
35 kDa
15 kDa
5 kDa

Figure 7:

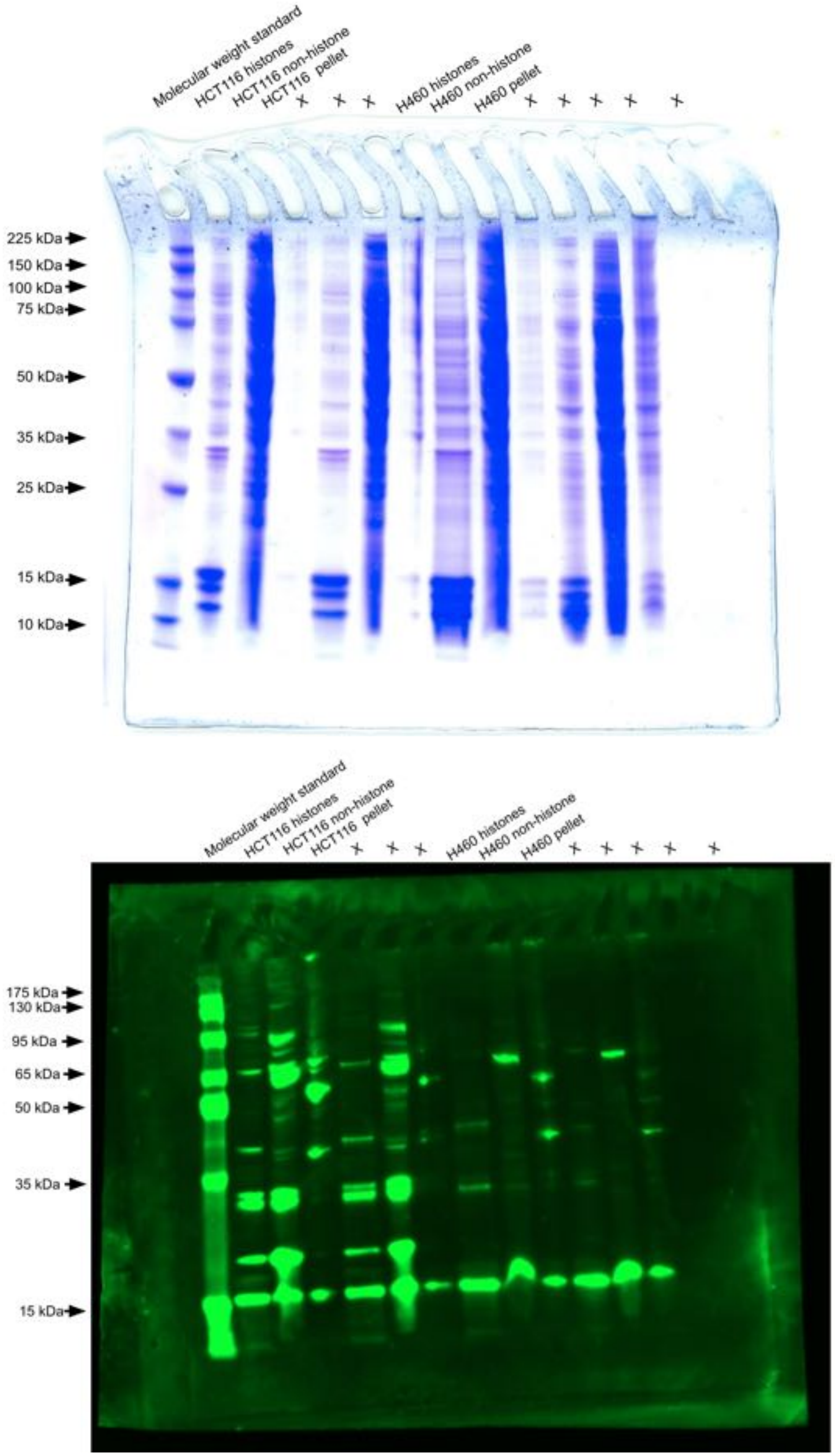
Molecular weight standard
HCT116 histones
HCT116 non-histone
HCT116 pellet
x
x
x
H460 histones
H460 non-histone
H460 pellet
x
x
x
x
x
225 kDa
150 kDa
100 kDa
75 kDa
50 kDa
35 kDa
25 kDa
15 kDa
10 kDa
Molecular weight standard
HCT116 histones
HCT116 non-histone
HCT116 pellet
x
x
x
H460 histones
H460 non-histone
H460 pellet
x
x
x
x
x
175 kDa
130 kDa
95 kDa
65 kDa
50 kDa
35 kDa
15 kDa

Figure 8:

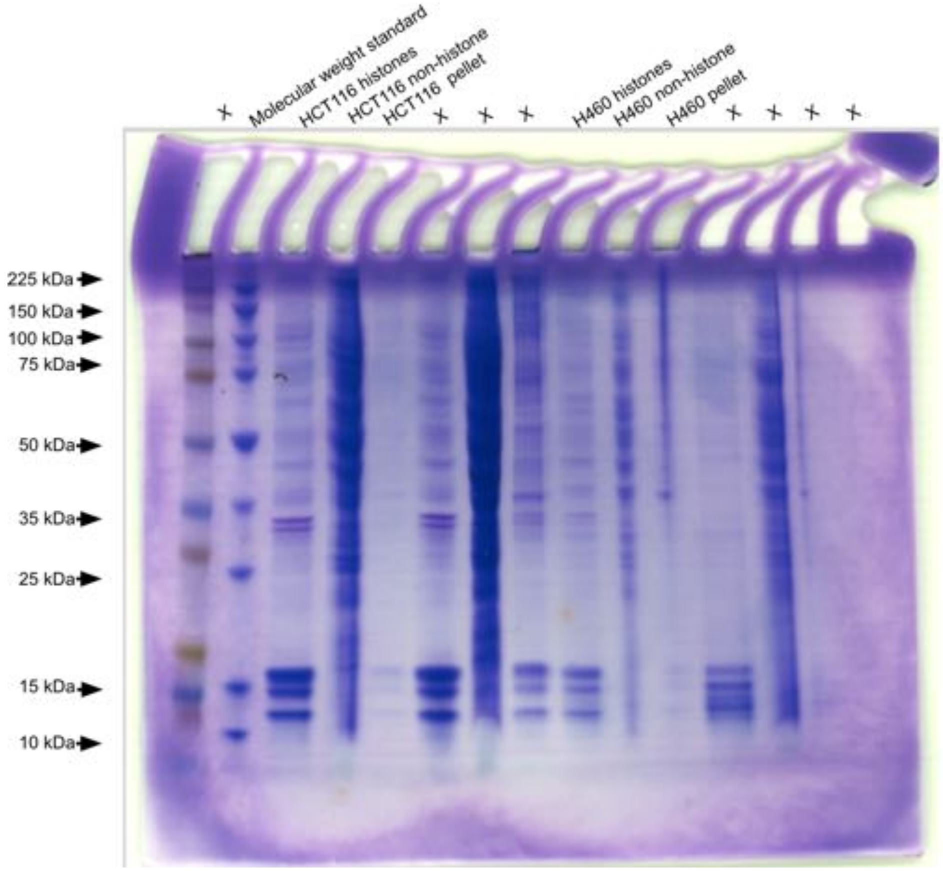
X
Molecular weight standard
HCT116 histones
HCT116 non-histone
HCT116 pellet
X
X
X
H460 histones
H460 non-histone
H460 pellet
X
X
X
X
225 kDa
150 kDa
100 kDa
75 kDa
50 kDa
35 kDa
25 kDa
15 kDa
10 kDa

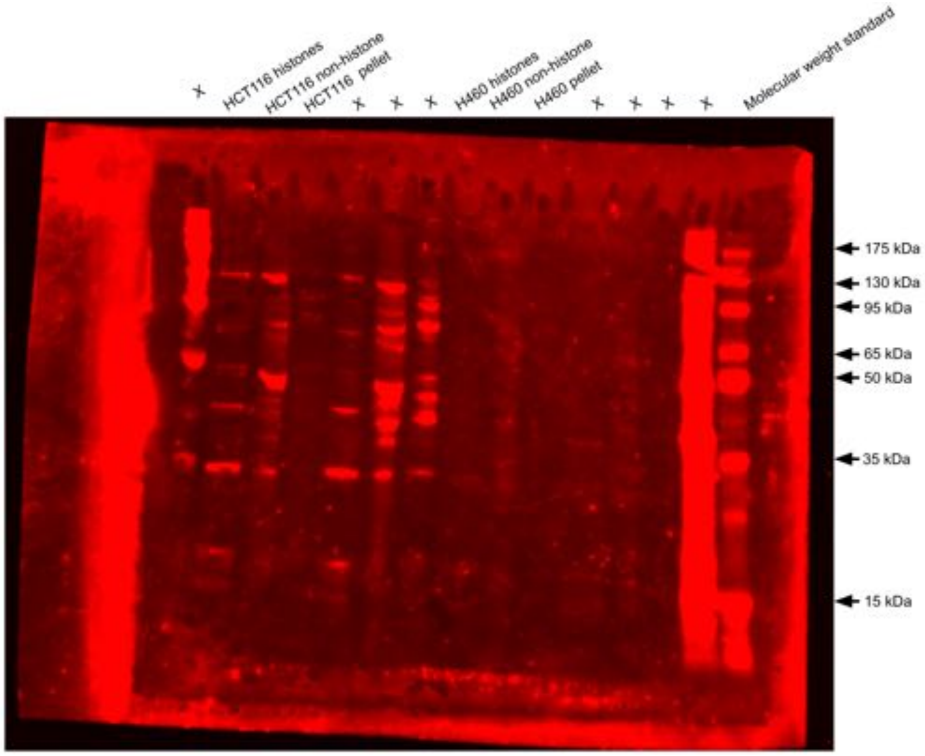
X
HCT116 histones
HCT116 non-histone
HCT116 pellet
X
X
X
H460 histones
H460 non-histone
H460 pellet
X
X
X
X
Molecular weight standard
175 kDa
130 kDa
95 kDa
65 kDa
50 kDa
35 kDa
15 kDa

Figure S2:

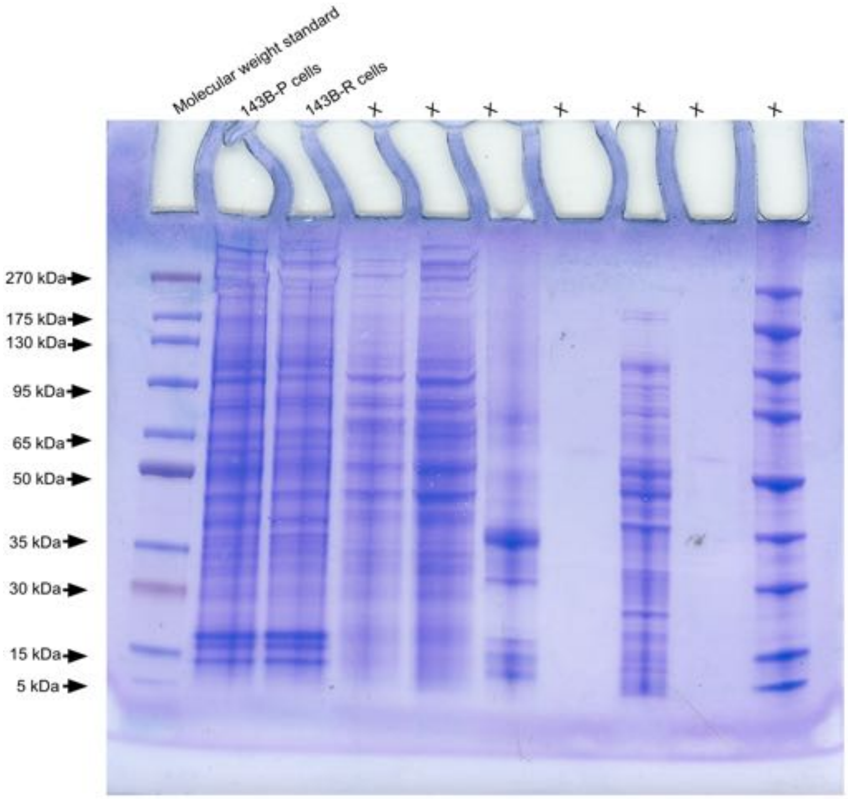
Molecular weight standard
143B-P cells
143B-R cells
x
x
x
x
x
x
x
270 kDa
175 kDa
130 kDa
95 kDa
65 kDa
50 kDa
35 kDa
30 kDa
15 kDa
5 kDa

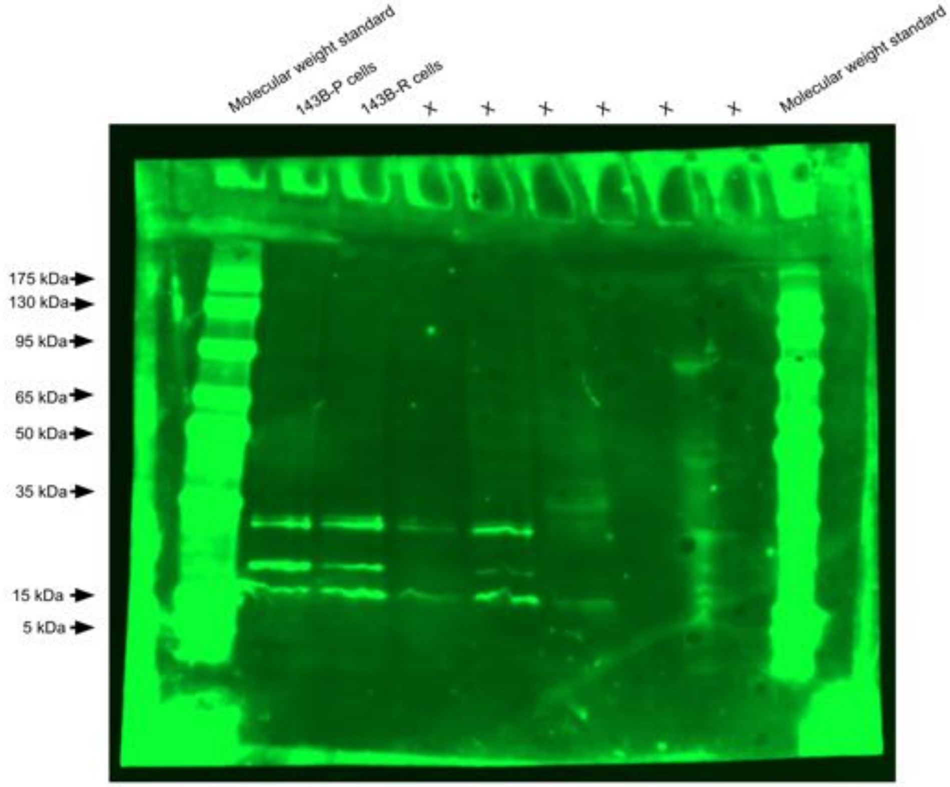
Molecular weight standard
143B-P cells
143B-R cells
x
x
x
x
x
x
Molecular weight standard
175 kDa
130 kDa
95 kDa
65 kDa
50 kDa
35 kDa
15 kDa
5 kDa

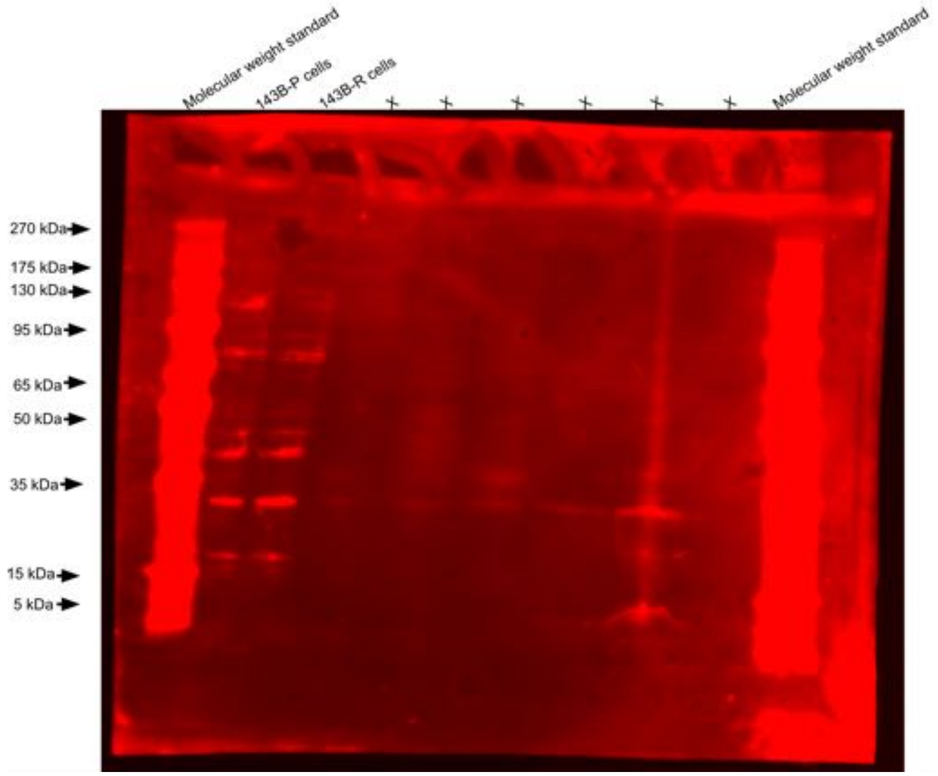

Molecular weight standard
143B-P cells
143B-R cells
X
X
X
X
X
X
Molecular weight standard
270 kDa
175 kDa
130 kDa
95 kDa
65 kDa
50 kDa
35 kDa
15 kDa
5 kDa

Figure S3:

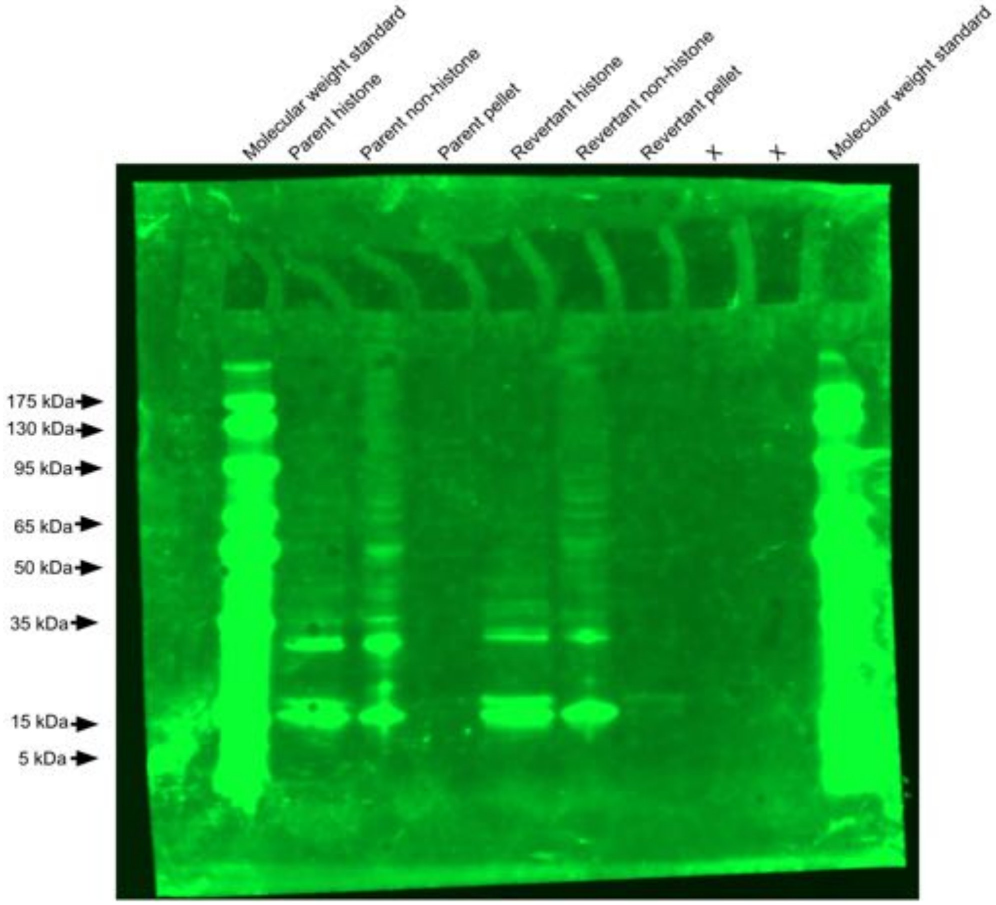

Molecular weight standard
Parent histone
Parent non-histone
Parent pellet
Revertant histone
Revertant non-histone
Revertant pellet
+
+
Molecular weight standard
175 kDa
130 kDa
95 kDa
65 kDa
50 kDa
35 kDa
15 kDa
5 kDa

Figure S4:

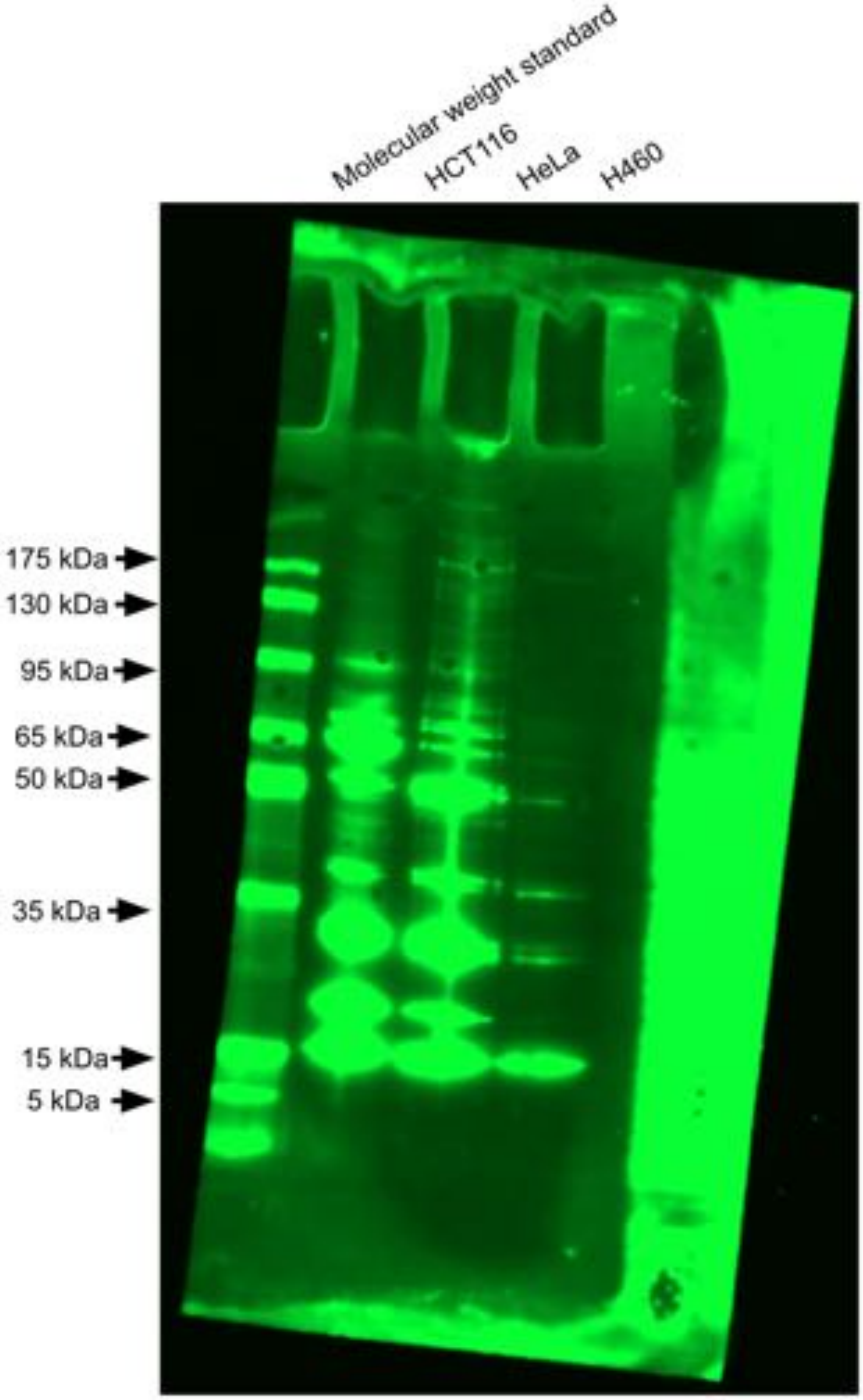
Molecular weight standard
HCT116
HeLa
H460
175 kDa
130 kDa
95 kDa
65 kDa
50 kDa
35 kDa
15 kDa
5 kDa

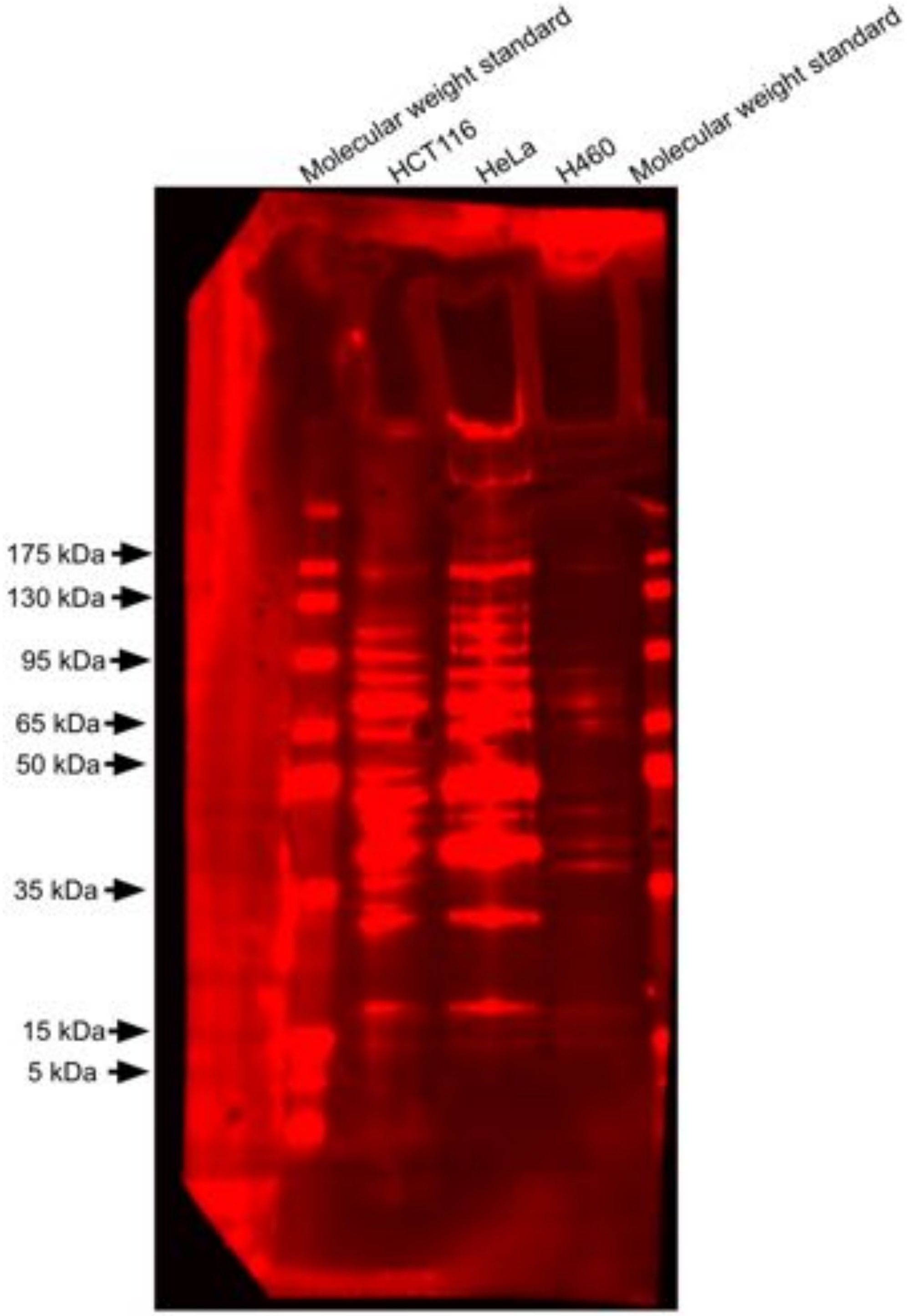
Molecular weight standard
HCT116
HeLa
H460
Molecular weight standard
175 kDa
130 kDa
95 kDa
65 kDa
50 kDa
35 kDa
15 kDa
5 kDa

Figure S5:

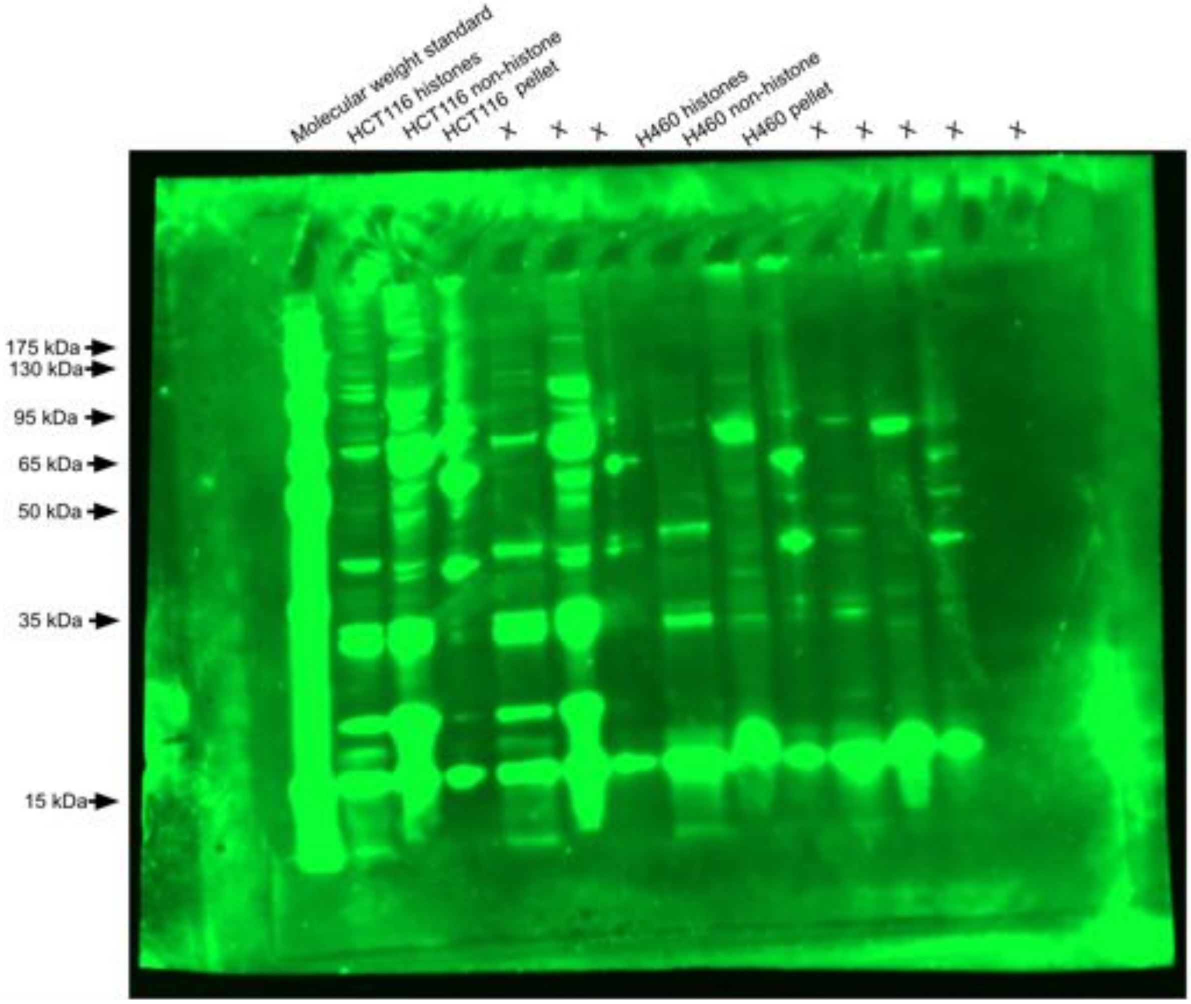
Molecular weight standard
HCT116 histones
HCT116 non-histone
HCT116 pellet
X
X
X
H460 histones
H460 non-histone
H460 pellet
X
X
X
X
X
175 kDa
130 kDa
95 kDa
65 kDa
50 kDa
35 kDa
15 kDa
